# Genome-wide association meta-analysis identifies five modifier loci of lung disease severity in cystic fibrosis

**DOI:** 10.1038/ncomms9382

**Published:** 2015-09-29

**Authors:** Harriet Corvol, Scott M. Blackman, Pierre-Yves Boëlle, Paul J. Gallins, Rhonda G. Pace, Jaclyn R. Stonebraker, Frank J. Accurso, Annick Clement, Joseph M. Collaco, Hong Dang, Anthony T. Dang, Arianna Franca, Jiafen Gong, Loic Guillot, Katherine Keenan, Weili Li, Fan Lin, Michael V. Patrone, Karen S. Raraigh, Lei Sun, Yi-Hui Zhou, Wanda K. O’Neal, Marci K. Sontag, Hara Levy, Peter R. Durie, Johanna M. Rommens, Mitchell L. Drumm, Fred A. Wright, Lisa J. Strug, Garry R. Cutting, Michael R. Knowles

**Affiliations:** 1 Assistance Publique-Hôpitaux de Paris (AP-HP), Hôpital Trousseau, Pediatric Pulmonary Department; Institut National de la Santé et la Recherche Médicale (INSERM) U938, Paris 75012, France; 2 Sorbonne Universités, Université Pierre et Marie Curie (UPMC) Paris 06, Paris 75005, France; 3 Division of Pediatric Endocrinology, Johns Hopkins University School of Medicine, Baltimore, Maryland 21287, USA; 4 AP-HP, Hôpital St Antoine, Biostatistics Department; Inserm U1136, Paris 75012, France; 5 Department of Genetics, University of North Carolina at Chapel Hill, Chapel Hill, North Carolina 27599, USA; 6 Marsico Lung Institute/UNC CF Research Center, School of Medicine, University of North Carolina at Chapel Hill, Chapel Hill, North Carolina 27599, USA; 7 Department of Epidemiology, Colorado School of Public Health, University of Colorado Denver, Anschutz Medical Center, Aurora, Colorado 80045, USA; 8 Children’s Hospital Colorado, Anschutz Medical Center, Aurora, Colorado 80045, USA; 9 Department of Pediatrics, School of Medicine, Anschutz Medical Center, Aurora, Colorado 80045, USA; 10 Division of Pediatric Pulmonology, Johns Hopkins University School of Medicine, Baltimore, Maryland 21287, USA; 11 McKusick-Nathans Institute of Genetic Medicine, Johns Hopkins University School of Medicine, Baltimore, Maryland 21287, USA; 12 Program in Genetics and Genome Biology, The Hospital for Sick Children, Toronto, Ontario, Canada M5G 0A4; 13 Program in Physiology and Experimental Medicine, The Hospital for Sick Children, Toronto, Ontario, Canada M5G 0A4; 14 Department of Statistical Sciences, University of Toronto, Toronto, Ontario, Canada M5S 3G3; 15 Division of Biostatistics, Dalla Lana School of Public Health, University of Toronto, Toronto, Ontario, Canada M5T 3M7; 16 Bioinformatics Research Center and Department of Biological Sciences, North Carolina State University, Raleigh, North Carolina 27695, USA; 17 Division of Pulmonary Medicine, Department of Pediatrics, Stanley Manne Research Institute, Northwestern University Feinberg School of Medicine, Ann and Robert Lurie Children’s Hospital of Chicago, Chicago, Illinois 60611, USA; 18 Department of Pediatrics, University of Toronto, Toronto, Ontario M5G 1X8, Canada; 19 Department of Molecular Genetics, University of Toronto, Toronto, Ontario, Canada M5S 1A8; 20 Department of Pediatrics, School of Medicine, Case Western Reserve University, Cleveland, Ohio 44106, USA; 21 Bioinformatics Research Center and Department of Statistics, North Carolina State University, Raleigh, North Carolina 27695, USA; 22 Department of Biological Sciences, North Carolina State University, Raleigh, North Carolina 27695, USA; 23 Department of Pediatrics, Johns Hopkins University School of Medicine, Baltimore, Maryland 21287, USA

## Abstract

The identification of small molecules that target specific *CFTR* variants has ushered in a new era of treatment for cystic fibrosis (CF), yet optimal, individualized treatment of CF will require identification and targeting of disease modifiers. Here we use genome-wide association analysis to identify genetic modifiers of CF lung disease, the primary cause of mortality. Meta-analysis of 6,365 CF patients identifies five loci that display significant association with variation in lung disease. Regions on chr3q29 (*MUC4/MUC20; P*=3.3 × 10^−11^), chr5p15.3 (*SLC9A3; P*=6.8 × 10^−12^), chr6p21.3 (HLA Class II*; P*=1.2 × 10^−8^) and chrXq22-q23 *(AGTR2/SLC6A14; P*=1.8 × 10^−9^) contain genes of high biological relevance to CF pathophysiology. The fifth locus, on chr11p12-p13 (*EHF/APIP; P*=1.9 × 10^−10^), was previously shown to be associated with lung disease. These results provide new insights into potential targets for modulating lung disease severity in CF.

## Abstract (web summary)

Cystic fibrosis imposes a decline in quality of life but new treatments are being developed that target specific *CFTR* variants. Here the authors identify five genome loci significantly associated with variation in disease severity in a meta-analysis, which may provide targets for individualized treatment of cystic fibrosis.

## Graphical abstract

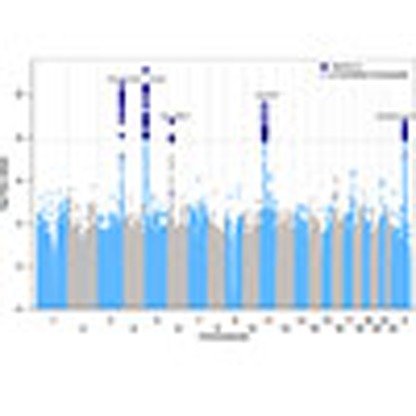

Cystic fibrosis (CF) affects ∼70,000 individuals worldwide and is caused by loss-of-function variants in *CFTR*. Although CF is regarded as a single-gene disorder, patients who have the same variants in *CFTR* exhibit substantial variation in severity of lung disease, of which >50% is explained by non-*CFTR* genetic variation^1^. The identification of small molecules that target specific *CFTR* variants has ushered in a new era of treatment for cystic fibrosis (CF)^2^, but optimal individualized treatment will require identification and targeting of disease modifiers.

The advent of large-scale genome-wide association studies (GWAS) and capability for imputation has made it possible to explore millions of polymorphisms in search of genetic determinants of phenotypic variation. Our previously reported GWAS in 3,444 CF patients led to identification of genome-wide significant single-nucleotide polymorphism (SNP) associations with lung disease severity in an intergenic region between *EHF* and *APIP* (chr11p13), as well as several additional suggestive loci (chr6p21.3 and chrXq22-q23)^3^. In other published candidate gene studies^4^, additional loci/genes have been reported to reach significance thresholds for the individual study, but many of these studies were based on relatively small sample sizes and/or limited phenotyping, and most have not been replicated, generating uncertainty as to their pathophysiological relevance.

In this manuscript, we have extended our study of CF gene modifiers by testing 2,921 additional patients from North America (*n*=1,699) and France (*n*=1,222). We use the same lung phenotype as in the previous GWAS (Consortium lung phenotype (KNoRMA))^5^, which allows for direct comparison of the lung function of CF patients irrespective of age and gender. A meta-analysis of both imputed and genotyped variants is reported that combines data from the new subjects with the previously reported GWAS, allowing for an unprecedented sample size of 6,365 CF patients and analysis of over 8 million variants. To maximize power, linear mixed models are used to allow for inclusion of CF-affected siblings. The combined analysis confirms a previous genome-wide association and identifies four new loci that contain genes with high biological relevance for pathophysiology of CF lung disease.

## Results

### Characteristics of patients in GWAS2 and GWAS1

The cohort study design and the demographic and clinical characteristics of GWAS2 subjects are detailed in Table 1 and Methods. In the combined GWAS1+2 data set, 99.8% of subjects were pancreatic exocrine insufficient (primarily defined by *CFTR* genotypes); 65.0% were p.Phe508del homozygotes; 95.5% were of European ancestry; and only 5.4% were diagnosed by newborn screening. GWAS1 subjects from three cohorts were genotyped on the same Illumina platform^3^, while GWAS2 included 10 subgroups defined by different combinations of site and Illumina genotyping platforms. These 13 subgroups had similar distributions of the lung disease phenotype (Supplementary Fig. 1).

### Genome-wide significance in five regions by meta-analysis

**Figure 1:**
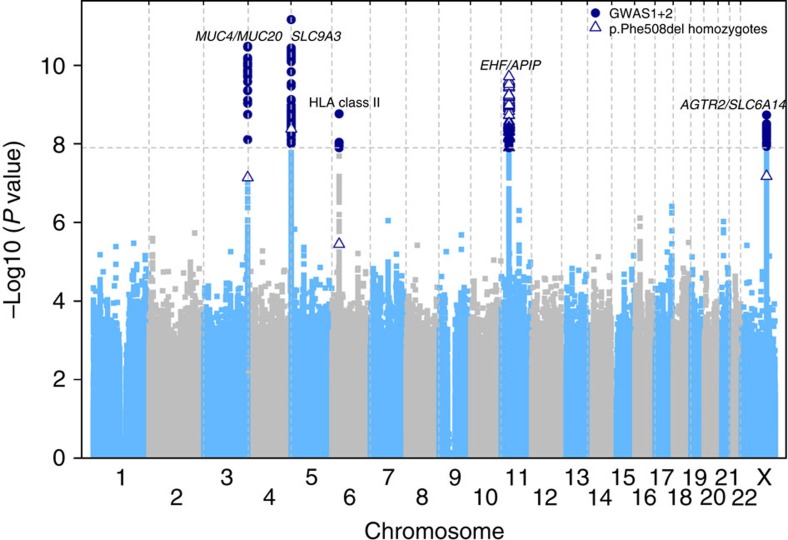
Genome-wide Manhattan plot of associations with the Consortium lung phenotype. Evidence from GWAS1+2 for all patients (closed circles) and for p.Phe508del homozygotes (open triangles). The horizontal dashed line represents the threshold for genome-wide significance (*P*<1.25 × 10^−8^). Genome-wide significance was achieved in five regions. The results from regions on chr5p15, chr11p12-p13 and chrXq22-q23 are from meta-analysis using a random effects model, and for chr3q29 and chr6p21 using a fixed effects model.

To account for potential effect size heterogeneity, a random effects meta-analysis association model^6^ (Methods) was applied across the 13 subgroups. Acknowledging that the power of fixed effects meta-analysis can be greater than that of random effects, even under heterogeneity^7^, we also performed fixed effect meta-analysis, and report loci exceeding significance thresholds by either random or fixed effects analysis. Analysis included 8,520,458 genotyped and imputed SNPs with minor allele frequency >0.005 and markers with imputation *r*^2^>0.3. Principal component-based stratification control was used (genomic control lambda=0.95) (Methods and Supplementary Figs 2 and 3). We considered loci with *P*<1.25 × 10^−8^ to be genome-wide significant, based on the standard for genome-wide significance (*P*=5 × 10^−8^)^8^ and multiplicity correction for *CFTR* genotype (all genotypes versus p.Phe508del homozygotes) and model (random versus fixed effects). Associations in five regions (chr3q29, chr5p15, chr6p21, chr11p12-p13 and chrXq22-q23) exceeded genome-wide significance (Fig. 1). Only the chr11p12-p13 locus provides significant evidence of interaction with *CFTR* (*P*=0.048, from a Wald test of the interaction term in the linear mixed model that is then meta-combined using the inverse variance-based weighting). This result corroborates our prior observation that association at this locus achieves a lower *P* value in p.Phe508del homozygous subjects than in all subjects^3^.

**Figure 2:**
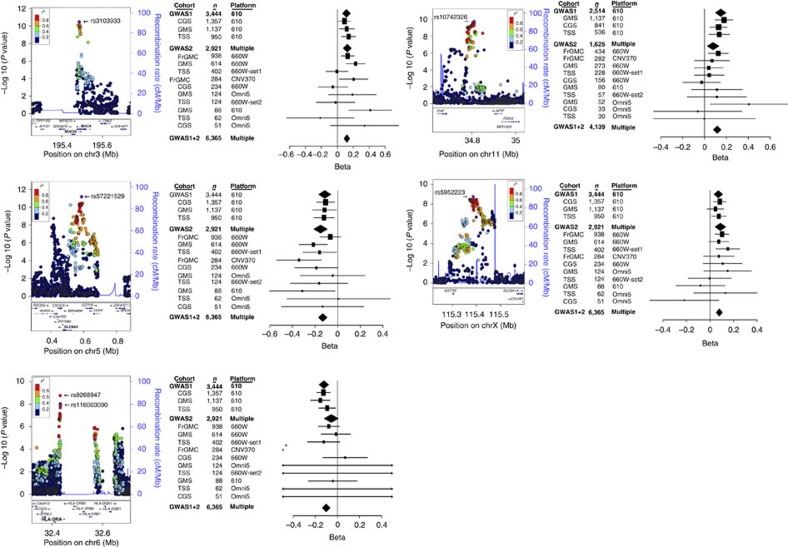
LocusZoom and forest plots for five regions with significant association in GWAS1+2. On the left side of the five panels are plots of the association evidence (build GRCh37, LocusZoom viewer) in the five genome-wide significant regions for all patients, except that chr11p12-p13 shows only p.Phe508del homozygotes. Colours represent 1000 Genomes EUR linkage disequilibrium *r*^2^ values with each SNP in column three of Table 2 (shown as purple diamonds and labelled with dbSNP ID). The purple diamond in the chr6 region denotes the SNP that has independent genotype confirmation, but the top imputed SNP is also indicated by a dbSNP ID (rs number). On the right side of the five panels are forest plots of the relative effect sizes for the most significant SNP in each of the 13 subgroups, ordered by size. Beta (coefficient) refers to the average change in Consortium lung phenotype for each copy of the minor allele. The size and shape of the squares are proportional to the weights used in the meta-analysis, and the line segments are 95% confidence intervals of each beta. The black diamonds represent summary data for GWAS1, GWAS2, and GWAS1+2. The asterisk on chr6 (HLA region) forest plot illustrates a beta (and confidence interval) for the FrGMC CNV370 subgroup of −19.9 (−35.4, −4.4). In addition, the beta (and confidence interval) for four other subgroups in the chr6 region are as follows: GMS Omni5, 0.87 (−19.5, 21.2); TSS 660W-set 2, −1.67 (−19.6, 16.3); TSS Omni5, 15.4 (−14.1, 44.8); and CGS Omni5, 1.7 (−23.8, 27.2).

Each of the regions contained at least one genotyped SNP achieving significance (Table 2), except for chr6p21.3 near *HLA-DRA*. However, five imputed SNPs that exceeded genome-wide significance at the *HLA-DRA* locus were directly genotyped in a subset of the sample and provided independent genotype confirmation (Methods). LocusZoom^9^ and effect size forest plots show the regional association evidence and the relative contribution from each of the 13 cohort by platform subgroups (Fig. 2). A formal test (Cochran’s *Q* test) revealed that only the *MUC4* locus has significant evidence of heterogeneity (*P*=0.04); the inconsistent association among subgroups may be due to the small sample size of some of these cohorts. All five regions all remained significant when restricted to 6,079 individuals determined by principal components to be of European ancestry (Supplementary Table 1, Supplementary Fig. 4). Complementary results from sub-setting the sample into a North American discovery (*n*=5,143) and French replication set (*n*=1,222) show genome-wide significance and independent replication in four of the five loci reported in Table 2. The remaining locus (chrX; *AGTR2/SLC6A14*) achieved suggestive evidence of association in the North American cohort with compelling evidence of replication in the French subjects (Methods and Supplementary Table 2).

### Conditioning on significant SNPs

Conditioning on the most significant SNP in the five regions that had genome-wide significance eliminated significant association in three regions (chr3q29; chr6p21; chrXq22-q23), but not for chr5p15 or chr11p12-p13. On chr5p15 (Supplementary Fig. 5a), conditioning on rs57221529 (located 5′ of *SLC9A3* and *CEP72*) revealed significantly associated SNPs 3′ of *SLC9A3* (near *AHRR*), suggesting a complex contribution by genes and/or regulatory domains in the region. Similar complex mechanisms may be at play on chr11p12-p13, as conditioning on rs10742326 (in *EHF*/*APIP* intragenic region) revealed significantly associated SNPs 3′ of *APIP* (Supplementary Fig. 5b).

### *CFTR* variants and lung function

As this study has the largest assembly of CF patients for modifier identification, we employed a combined test for association between *CFTR* variants and lung function^10^, using the maximal unrelated sample of patients from GWAS1+2 (*n*=5,762). Summing the association test statistics (the square of the association *Z*-statistic) for each SNP in/near *CFTR* enabled capture of the combined effect of disease-causing mutations (Methods). Overall, this gene-based *(CFTR)* statistic showed that *CFTR* and lung disease severity are associated (*P*=0.0043, from a permutation-based test that is then meta-combined using Stouffer's *Z*-score method), but the evidence is very modest compared to the evidence for multiple modifier loci reported here. As expected, this association with lung disease was not present when we restricted analysis to the p.Phe508del homozygotes (*n*=3,815; *P*=0.1604, from a permutation-based test that is then meta-combined using Stouffer's *Z*-score method).

### eQTLs for significant SNPs

To determine whether the most significantly associated GWAS SNPs show evidence as expression quantitative trait loci (eQTLs) in lung or immune cells, we examined three available databases for eQTLs (Genotype-Tissue Expression, GTEx, http://www.gtexportal.org/home/; University of Chicago SNP and CNV Annotation Database, SCAN, http://eqtl.uchicago.edu/cgi-bin/gbrowse/eqtl/; University of North Carolina at Chapel Hill seeQTL, http://www.bios.unc.edu/research/genomic_software/seeQTL/). We considered eQTL evidence for each of the top-ranked GWAS SNPs at each locus, and for other local SNPs with *r*^2^ (linkage disequlibrium, LD)>0.6 with the top-ranked GWAS SNPs. The most significant eQTLs were noted at the chr6 (human leukocyte antigen (HLA) Class II) locus (*DRB1* and *DRB5*) and the chr11 locus (*APIP*). The nominal *P* values from association testing in a linear regression model for eQTLs at other loci were modest (*P*>10^−5^), and of uncertain biological significance (Supplementary Table 3).

### Comparison of GWAS1+2 with prior association studies

We also compared the results of the GWAS1+2 analysis with variants in 30 candidate genes or loci previously reported to associate (nominal *P*<0.05) with some aspect of CF pulmonary disease phenotype (Supplementary Table 4). For previously reported candidate gene SNP associations, only those in *SLC9A3* demonstrated significance (after correction for multiplicity of replication testing) in GWAS1+2, although others trended towards nominal significance. It is important to recognize that none of these studies used precisely the same lung phenotype as GWAS1+2, that each study involved substantially fewer subjects than the 6,365 individuals studied here, and that other SNPs in these candidate regions were not assessed here. Likewise, homogeneity of non-genetic factors such as healthcare delivery might have enabled discovery of modifier associations in some studies that are not detectable in the heterogeneous populations aggregated for this study.

## Discussion

Given the rarity of Mendelian disorders such as CF, it is not possible to accrue the large population of samples achieved for common complex traits^11^,^12^. The uniform genetic aetiology of Mendelian disorders can minimize phenotypic and genetic heterogeneity, and we take advantage of this uniformity to identify five genome-wide significant loci associated with the severity of lung disease in CF. Despite minimizing genetic heterogeneity, some residual effect of *CFTR* sequence variation on variation in lung function remained evident, suggesting a contributing but modest role compared to the modifiers. On the basis of the calculated beta-coefficients, the potential effect sizes of the SNPs of interest are estimated to be clinically relevant. The largest absolute beta-coefficient value (0.13) for *SLC9A3*/rs57221529 is equivalent to a change in forced expiratory volume at one second (FEV_1_) of 190 ml (4.64% predicted) for males and 130 ml (3.98% predicted) for females based on extrapolation from 18-year-old White subjects with lung function and height both at the 50th percentile for individuals with CF^13^,^14^. Likewise, the smallest absolute beta-coefficient value (0.08) for *AGTR2*/*SLC6A14*/rs5952223 is equivalent to a change in FEV_1_ of 110 ml (2.68% predicted) for males and 80 ml (2.45% predicted) for females.

The five associated loci contain genes with pathophysiological relevance for lung function in CF, and genes in these regions are expressed in lung^3^,^15^,^16^,^17^,^18^,^19^. Identification of the gene or genes responsible for the modifying effect of each locus will require functional study; however, each locus contains genes of compelling biologic plausibility based on extensive understanding of CF pathophysiology. *MUC4* and *MUC20* located at chr3q29 encode membrane-spanning ‘tethered’ mucins on ciliated airway mucosal surfaces^18^,^20^,^21^ that contribute to the periciliary brush layer, and prevent mucus penetration into the periciliary space^22^. Further, MUC4 and MUC20 are present in airway mucus, reflecting secretion and/or shedding from the airway epithelium^18^, and likely play a role in mucociliary host defense. *SLC9A3* on chr5 is the cation proton antiporter 3 (*NHE3*) involved in pH regulation and epithelial ion transport^23^,^24^. Knockout of *SLC9A3* alleviated intestinal obstruction commonly observed in the mouse model of CF^25^, and a subsequent hypothesis-driven genome-wide association study identified *SLC9A3* as a modifier of neonatal intestinal obstruction in humans with CF^26^. Candidate gene studies in the Canadian pediatric CF population have shown *SLC9A3* to be pleiotropic for disease severity in multiple affected organs^17^,^27^. The broadness of the associated region does not exclude *EXOC3* (telomeric, and just downstream of *SLC9A3*), a component of the exocyst complex that is involved in post Golgi trafficking and specification of membrane surfaces, including those in epithelial cells^28^. Further, the two more centromeric genes, *CEP72* and *TPPP*, have been implicated in microtubule function. Microtubule disturbance has been reported in CF cells^29^ and a microtubule-associated gene, *DCTN4* has been implicated in a CF-lung related phenotype of onset of *Pseudomonas aeruginosa (P. aeruginosa)* infection^30^. The HLA Class II region on chr6 has been associated with asthma, variation in lung function in the non-CF populations, and CF lung disease^19^,^31^,^32^, along with susceptibility to allergic bronchopulmonary aspergillosis^33^,^34^,^35^. Moreover, HLA Class II pathways have been recently associated with CF lung disease and age-of-onset of persistent *P. aeruginosa* in a large gene expression association study^36^. The chrX locus contains *AGTR2* and *SLC6A14* and either gene, or possibly both, could modify lung disease. *AGTR2*, the angiotension type II receptor, has been implicated in a variety of pulmonary functions including mediating signalling in lung fibrosis^15^, regulating nitric oxide synthase expression in pulmonary endothelium^37^, and has recently been described as a therapeutic target for lung inflammation^38^. *SLC6A14* encodes an amino-acid transporter and variants in its 5′-regulatory region have been reported to modify risk for neonatal intestinal obstruction^26^, lung disease severity, and age at first *P. aeruginosa* infection in individuals with CF under 18 years of age^17^. The chr11 locus containing *EHF* and *APIP* was reported to be associated with CF lung disease in p.Phe508del homozygotes in an earlier GWAS^3^ and this association persists in this report. This EHF transcription factor is reported to modify the CF phenotype by influencing p.Phe508del processing^16^, and to modulate epithelial tight junctions and wound repair^39^. APIP is a methionine salvage pathway enzyme known to be associated with both apoptosis and systemic inflammatory responses^40^,^41^. Examination of the most significantly associated GWAS SNPs revealed eQTLs for genes at the HLA Class II locus (*DRB1* and *DRB5*) and *APIP* (chr11). These findings are congruent with lung disease severity being mediated through differential gene expression (eQTLs), but additional studies will be required to clarify this possibility for each locus.

Collectively, this study suggests new mechanistic insight into ameliorating lung disease progression in individuals with CF. Functional analysis of associated SNPs and genes at each modifier locus could identify novel targets for treating CF. For example, BCL11A, a key transcription factor for fetal haemoglobin expression, is a modifier of beta thalassaemia and sickle cell disease^42^,^43^. Identification of a common variant associated with fetal haemoglobin level that alters *BCL11A* expression provides a therapeutic rationale for targeting this modifier of haemoglobinopathies^44^. The evolving paradigm for individualized therapy in CF involves small molecules targeting specific *CFTR* variants^2^,^45^. The discovery of modifier loci that are strongly associated with severity of CF lung disease provides an opportunity to enhance individualized treatment in CF.

## Methods

### Recruitment

The recruitment in North America was performed through three independent groups (GMS, CGS and TSS studies; Table 1)^1^,^46^,^47^. In brief, the majority of the GMS subjects were recruited as unrelated extremes-of-phenotype (lung disease severity, mild versus severe), whereas a subset of patients were recruited as a population-based sample at the Children’s Hospital of Denver, Wisconsin, and Boston, and also from the multicenter study UNC/CWRU GMS cohort (Table 1). The CGS patients were recruited from the Canadian population of CF patients. The TSS patients were recruited based on having a surviving affected sibling. Informed consent was obtained from each participant of the study. Studies were approved by institutional review boards at participating sites and include: Committee on Clinical Investigation, Boston Children's Hospital; Institutional Review Board at Children’s Hospital of Wisconsin; Colorado Multiple Institutional Review Board; Johns Hopkins School of Medicine eIRB2 (Committee: IRB-3); Research Ethics Board of The Hospital for Sick Children; Biomedical Institutional Review Board, Office of Human Research, University of North Carolina at Chapel Hill; and University Hospitals Case Medical Center, Institutional Review Board for Human Investigation. In France, patients were recruited from 48 CF centers, and phenotypic information was available for 2,898 patients older than 6 years of age; further, only patients with both parents born in European countries were considered (*n*=2,627). Only patients with documented pancreatic insufficiency or having two severe *CFTR* mutations were further considered. Written informed consent was obtained from adults, and for patients<18 years old there was consent from parents or guardians for participation in the study. The study was approved by the French ethical committee (CPP n°2004/15) and the information collection was approved by CNIL (n°04.404).

### Lung disease severity phenotyping

In CF, FEV_1_ is recognized as producing the most clinically useful measurements of lung function and a known predictor of survival^46^,^47^. However, comparison of disease severity by FEV_1_ across a broad range of ages is confounded by the decline in FEV_1_ over time in CF patients, and by mortality attrition. In brief, we calculated average age-specific CF percentile values of FEV_1_ for each patient using three years of data in patients 6 years or older, using the Kulich-derived US (national)^3^ or French (national)^48^ CF percentiles (relative to other CF patients of the same age, sex and height), and adjusted for mortality^5^. The resulting quantitative phenotypes were distributed as expected, based on ascertainment. This quantitative phenotype was also highly correlated with the Schluchter survival phenotype (*r*^2^=0.91), when compared for the GMS patients^47^.

### Genotyping and quality control

Genotyping used Illumina platforms, including: CNV370 (*n*=284 samples; FrGMC); 610 (*n*=3,532 samples; primarily GWAS1); 660W (*n*=2,312 samples); and Omni5 (*n*=237 samples). Genotype calling was performed using GenomeStudio V2011.1. Position and annotation information is based on hg19.

For the 660W platform, the total number of probes was 655,214 (64,569 were CNV probes) and 55 of the SNPs had no genotype calls for any of the samples. The 2,312 samples were composed of 938 (FrGMC)+614 (GMS)+234 (CGS)+402 (TSS 660W-set1)+124 (TSS 660W-set2) (Supplementary Fig. 1).

For the Omni5 platform, the total number of probes was 4,301,332 (961 of the SNPs had no genotype calls for any of the samples). The 237 samples were composed of 124 (GMS)+51 (CGS)+62 (TSS) (Supplementary Fig. 1).

For GWAS2, some samples (*n*=24) were genotyped on both 660W and Omni5 platforms and these duplicates showed >98% concordance. There were also 40 quality control duplicates of GWAS2 and GWAS1 samples, and those duplicates also showed high concordance. Samples with a call rate <98%, or sex discordance were excluded. Unintentional duplicates of GWAS2 and GWAS1 samples, and individuals missing a Consortium lung phenotype were also excluded. After exclusions, 2,921 GWAS2 samples remained for the analysis. There were 473,514 SNPs present on both the 660W and Omni5 platforms.

### Imputation

MaCH/Minimac software (http://www.sph.umich.edu/csg/abecasis/MACH/index.html) was used for phasing and imputing the genotyped data. Phase I, Version 3 haplotype data from 1000 Genomes project (ftp://ftp-trace.ncbi.nih.gov/1000genomes/ftp/release/20110521/) was used as the reference. Approximately 5% of our patients were deemed as not of European ancestry by principal components (Supplementary Fig. 4), so all 1000 Genomes reference samples were included. Samples were imputed separately by genotyping platform and site. Genotyped SNPs with a low minor allele frequency and low call rate were excluded prior to imputation. Imputed SNPs with a MaCH quality score *r*^2^<0.30 were excluded from the analysis.

### Genotype verification of imputed SNPs

At the *HLA* locus, six SNPs (rs140348826, rs143609473, rs145621799, rs145182993, rs116003090 and rs115367740) in high linkage disequilibrium with the SNP (rs9268947) with the lowest *P* value (in the meta-analysis using inverse variance-based weighting) reached genome-wide significance in the meta-analysis. The allele assignments of all seven SNPs were imputed, although some of these SNPs were genotyped on one or more platforms. To evaluate the accuracy of the imputation of these seven SNPs we performed two analyses. First, we compared the genotyping results of one of these SNPs (rs116003090 included on the Omni5 platform) in 374 individuals. Concordance between the imputed allele assignments for this SNP and the 374 genotypes of the SNP was 100%. Second, we obtained whole-genome sequencing results from 94 Canadian CF individuals who were subjects in the meta-GWAS analysis. These 94 subjects had all seven SNPs phased by imputation based on genotyping on the 610 platform. Five of the seven SNPs were called with high confidence in the whole-genome sequence. Of these five SNPs, the concordance rate between the sequencing results and the imputation (rounded to the nearest integer) was 98% (rs143609473), 99% (rs140348826 and rs145182993) and 100% (rs116003090 and rs115367740). These results independently verify the quality of the imputation of five of the seven SNPs at the HLA locus that exceeded genome-wide significance. The remaining imputed SNP (rs9268947) has the lowest *P* value in the region, but lacks independent genotype confirmation. Thus, rs116003090, the SNP with the most extensive and best concordance with independent genotyping, was selected as the representative associated SNP from the *HLA* region.

### Estimating population structure with principal components

Eigensoft (http://www.hsph.harvard.edu/alkes-price/software/) was utilized to obtain principal components to serve as covariates to adjust for population stratification. Genotyped SNPs common in all platforms with a high minor allele frequency and low linkage disequilibrium were included. Principal components were generated separately for each subgroup. To account for relatives, a maximal set of unrelated individuals were first identified by choosing a random member from each family for principal component analysis. Principal components for the held-out members were then inferred from the loadings. The Tracy–Widom statistic, as computed in Eigensoft, was used to evaluate the statistical significance of each principal component. Individuals were judged to have European ancestry if principal components 1 and 2 fell within a mean±6 s.d. rectangle formed using principal components from the HapMap3 European data set (Supplementary Fig. 4).

### Association testing

Genome-wide association was conducted in each of the 13 subgroups. Linear regression was used for the GMS and FrGMC samples; linear mixed modelling was used in the TSS and CGS subgroups to allow the inclusion of siblings in the analysis, accounting for familial correlation by including a random effect for each family. For each subgroup, we tested for interaction between *CFTR* and modifier loci by adding an indicator variable for p.Phe508del homozygotes versus others then performing a meta-analysis on the interaction term. Within each subgroup analysis, we included sex and significant principal components (based on the Tracy–Widom statistic *P*<0.05) as covariates in the association models for each SNP. The SNP was coded additively in the model. The SNP-specific beta coefficient and s.e. from each subgroup analysis were used as input for the meta-analysis. Genome-wide significance was defined (*P*≤1.25 × 10^−8^)^49^.

### Alternate study design

Subjects were divided into two pools to evaluate our results using a replication-based study design. The North American subjects (*n*=5,143) constituted a Discovery sample and the French subjects (*n*=1,222) served as a Replication sample, a strategy employed in a prior publication^3^. Under this design, four of the five loci (chr3q29, chr5p15, chr6p21, and chr11p12-p13) achieved genome-wide significance (*P*<5 × 10^−8^)^49^ in the North American sample with independent replication in the French subjects (*P*=8.7 × 10^−5^, 0.003, 0.054 and 0.003, respectively; Supplementary Table 2). The *AGTR2/SLC6A14* locus achieved suggestive evidence of association in the North American cohort (*P*=9.8 × 10^−7^) with compelling evidence of replication in the French subjects (1.8 × 10^−4^). Note that one locus (chr11p12-p13) had previously achieved genome-wide significance in the discovery sample and replication sample in a study of North American subjects^3^. In the current study, the *EHF/APIP* locus retains genome-wide significance in subjects from North America and the association is replicated in the 1,222 French subjects (*P*=0.003).

### *CFTR* gene-based testing

To assess whether variability in lung disease is associated with *CFTR* genotype within our sample of individuals with severe (pancreatic insufficient) mutations, we used gene-based association analysis. From our imputed SNP set, there were 539 SNPs annotated to *CFTR* (+/− 10 kb). We constructed a gene-based test statistic in the maximal set of unrelated individuals in each of the 13 subgroups (*n*=5,762), and restricted to this unrelated set for ease of permutation. To compute the gene-based test statistic, we obtained residuals by regressing the Consortium lung phenotype (KNoRMA) and each SNP genotype on the principal components and sex. The association test statistic between the residualized phenotype and residualized SNP was computed, squared and then summed across the 539 SNPs. We used permutation to obtain subgroup-specific permuted statistics, permuting the residualized phenotype 10,000 times to obtain 10,000 gene-based sum statistics under the null hypothesis of no association to obtain *P* values for each subgroup, which preserves the linkage disequilibrium structure in the region. The *P* value was calculated as the proportion of sum statistics from the 10,000 permutations that were more extreme than the observed *CFTR* sum statistic. Stouffer’s *Z*-score method was used to combine *P* values from each subgroup and weight them by their respective sample sizes.

### Phenotype variation attributable to association

To estimate the proportion of variability in Consortium lung phenotype that is explained by our five significant regions, we conducted an association analysis using the maximum set of unrelated individuals in each subgroup, using the five SNPs from Table 2 in the regression model. Then we calculated an average *r*^2^, weighted by sample size.

### eQTL assessment for most significantly associated GWAS SNPs

We searched for eQTLs for the top-ranked SNP in each of five loci, and for local SNPs in *r*^2^ (LD)>0.6 with the top-ranked GWAS SNPs, using three publicly available databases (Genotype-Tissue Expression, GTEx, lung and whole blood; University of North Carolina at Chapel Hill seeQTL, HapMap LCL and Zeller monocyte; University of Chicago SNP and CNV Annotation Database, SCAN, HapMap CEU LCL). Genes and transcripts within 100 kb of the top-ranked SNPs in five loci were included. Results are presented as nominal *P* values for each region, except *Q* values are given for the results from UNC seeQTL (Supplementary Table 3).

### GWAS1+2 comparison with prior candidate associations

A literature search was conducted to identify previously published reports of variants associated with some aspect of pulmonary function with a significance value of *P*<0.05, from association testing in a regression model, (Supplementary Table 4). The gene suggested as likely responsible for the associations was identified, and the chromosome on which it resides. For SNPs, the rs number was identified, along with other nomenclature (aliases) for the variant. A variety of phenotyping methods had been used in these studies, ranging from X-ray and clinical scores to objective measures of lung function, both cross-sectional and longitudinal. The associating phenotypes are summarized under ‘Phenotypes Tested’ and the reported *P* value. The number of subjects analysed and the publication were defined and the *P* values for any of these SNPs that were tested in this study are given for all subjects (All) and for p.Phe508del homozygotes alone.

## Additional information

**How to cite this article:** Corvol, H. *et al*. Genome-wide association meta-analysis identifies five modifier loci of lung disease severity in cystic fibrosis. *Nat. Commun.* 6:8382 doi: 10.1038/ncomms9382 (2015).

## Supplementary Material

Supplementary Information Supplementary Figures 1-5, Supplementary Tables 1-4 and Supplementary References.

## Figures and Tables

**Table 1:** Characteristics of patients enrolled in GWAS2 and GWAS1 by the International Cystic Fibrosis Gene Modifier Consortium.

				**Age (years)**						
	**Lead Institution(s)**	**Design**	**Subjects** ***n***	**Mean (±s.d.)**	**Range**	**Male** ***n*** **(%)**	European* ***n*** **(%)**	**p.Phe508del/p.Phe508del** ***n*** **(%)**	**Pancreatic exocrine insufficient** ***n*** **(%)**	**Subjects identified by NBS** ***n*** **(%)**
*GWAS2*										
French CF Gene Modifier Consortium (FrGMC)	University of Pierre and Marie Curie, Inserm U938	Population based	1,222	21.0 (9.2)	6.0–57.6	627 (51.3)	1,211 (99.1)	716 (58.6)	1,222 (100.0)	63 (5.2)
Genetic Modifier Study (GMS)	University of North Carolina/ Case Western Reserve University	Extremes of phenotype	469	25.8 (10.9)	7.9–62.2	256 (54.6)	407 (86.8)	191 (40.7)	467 (99.6)	3 (0.01)
		Population-based†	357	20.3 (10.0)	6.6–60.2	191 (53.5)	336 (94.1)	214 (59.9)	357 (100.0)	137 (38.4)
										
Canadian Consortium for Genetic Studies (CGS)	Hospital for Sick Children	Population based‡	285	13.0 (7.6)	6.4–40.0	150 (52.6)	268 (94.0)	189 (66.3)	282 (98.9)	0 (0.0)
Twins and Sibs Study (TSS)	Johns Hopkins University	Family based and population based§	588	15.8 (10.3)	6.0–56.0	305 (51.9)	533 (90.6)	315 (53.6)	583 (99.1)	54 (9.2)
										
	Summary GWAS2		2,921	19.9 (10.4)	6.0–62.2	1,529 (52.3)	2,755 (94.3)	1,625 (55.6)	2,911 (99.7)	257 (8.8)
										
*GWAS1*										
	Summary GWAS1||		3,444	19.2 (8.5)	6.0–56.0	1,839 (53.4)	3,324 (96.5)	2,514 (73.0)	3,444 (100.0)	84 (2.4)
										
*GWAS1+2*										
	Summary GWAS1+2		6,365	19.5 (9.4)	6.0–62.2	3,368 (52.9)	6,079 (95.5)	4,139 (65.0)	6,355 (99.8)	341 (5.4)

GWAS, genome-wide association study; NBS, newborn screening.

^*^On the basis of Eigenstrat principal components analysis and closeness to CEU.

^†^Includes patients enrolled into studies at Children’s Hospitals in Boston, Colorado and Wisconsin, and through UNC/CWRU; includes 3 two-sibling families.

^‡^Includes 13 two-sibling families and 1 three-sibling family, plus 256 singletons.

^§^148 two-sibling families, 4 three-sibling families, plus 280 singletons.

^||^Wright *et al*.^3^.

**Table 2:** Genome-wide significant association results for GWAS1+2.

**Chr**	**3**	**5**	**6**	**11**	**X**
Nearby gene(s)	*MUC4/MUC20*	*SLC9A3*	*HLA-DRA*	*EHF/APIP*	*AGTR2/SLC6A14*
SNP*	rs3103933	rs57221529	rs116003090†	rs10742326	rs5952223
Base pair‡	195,485,440	586,624	32,434,850	34,810,010	115,386,565
Minor allele§	A	G	C	A	T
Major allele§	G	A	G	G	C
Minor allele frequency||	0.37	0.2	0.31	0.42	0.28
GWAS1+2 all beta coefficient¶	0.12	−0.13	−0.1	0.09	0.08
GWAS1+2 p.Phe508del/p.Phe508del beta coefficient¶	0.12	−0.13	−0.09	0.12	0.09
*P* value, GWAS1+2 all#	3.3 × 10^−11^	6.8 × 10^−12^	1.2 × 10^−8^	4.8 × 10^−9^	1.8 × 10^−9^
*P* value, GWAS1+2 p.Phe508del/p.Phe508del#	7.6 × 10^−8^	3.4 × 10^−8^	3.6 × 10^−5^	1.9 × 10^−10^	1.3 × 10^−5^
Analysis with maximum significance	GWAS1+2 all**	GWAS1+2 all	GWAS1+2 all**	GWAS1+2 p.Phe508del/p.Phe508del	GWAS1+2 all
SNP, genotyped††	rs2246901	rs3749615	rs2395185	rs10466455	rs5905376
*P* value, genotyped††	1.3 × 10^−10^	2.2 × 10^−9^	1.5 × 10^−7^	1.3 × 10^−9^	3.3 × 10^−9^

GWAS, genome-wide association study; SNP, single-nucleotide polymorphism.

^*^SNP IDs are from 1000 Genomes Project (Phase I, Version 3). The proportion of variability in Consortium lung phenotype explained by the five SNPs is 0.05.

^†^rs116003090 is an imputed SNP that was genotyped on a subset of subjects (*n*=374) for independent genotype confirmation.

^‡^Genome Reference Consortium Human Build 37 (GRCh37).

^§^Major/minor alleles indexed to 1000 Genomes Project.

^||^Minor allele frequencies are listed for all GWAS1+2.

^¶^Beta-coefficients refer to the average change in Consortium lung phenotype for each copy of the minor allele.

^#^Meta-analysis *P* values based on a random effects model,

^**^with the exceptions of rs3103933 and rs116003090, which were based on a fixed effects model.

^††^Most significant SNP genotyped on all platforms; P values based on called genotypes.
