# Supplementary material for: Genome-wide association meta-analysis identifies five modifier loci of lung disease severity in cystic fibrosis
Source: Nat Commun. 2015 Sep 29;6:8382. doi: 10.1038/ncomms9382 (PMC4589222; doi:10.1038/ncomms9382)
Supplement: Supplementary Information — Supplementary Figures 1-5, Supplementary Tables 1-4 and Supplementary References. [file ncomms9382-s1.pdf]

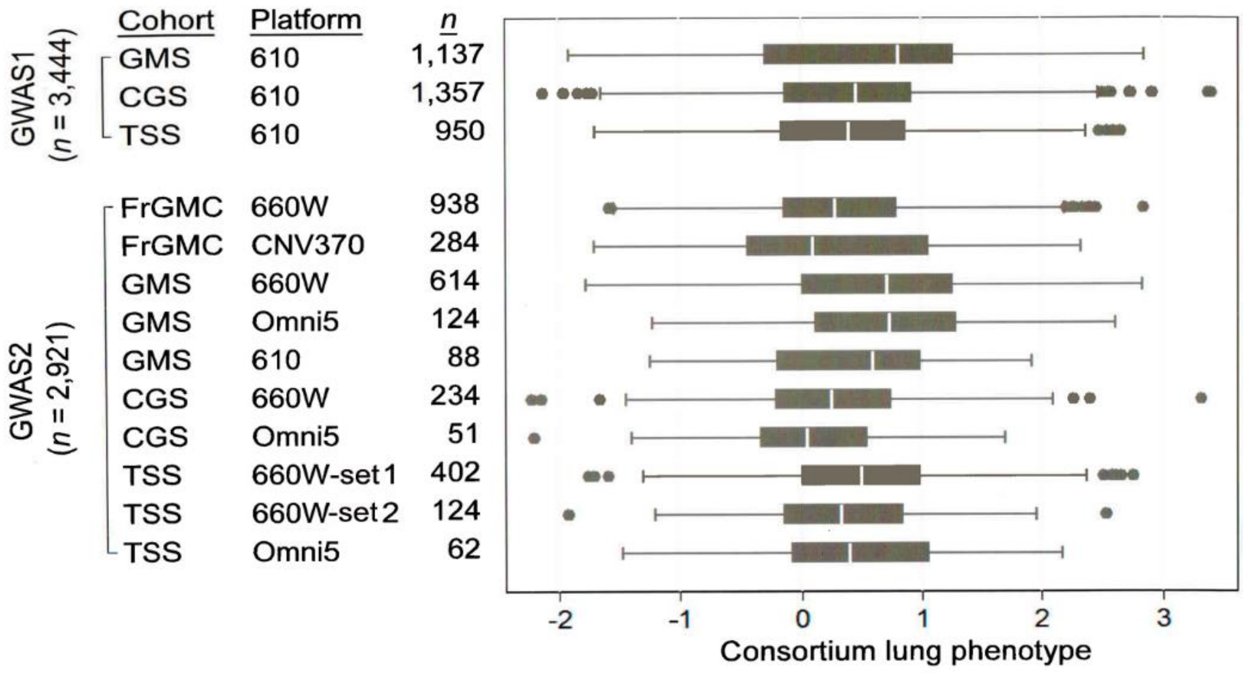

Supplementary Figure 1

Box and whiskers plots of the Consortium lung phenotype for 13 subgroups defined by combination of site and genotyping platform.

The borders of the box are the 1st and 3rd quartiles, and the band inside the box is the median. The interquartile range (IQR) is the 1st quartile subtracted from the 3rd quartile, and the whiskers are ±1.5*IQR. Values not included between the whiskers are plotted as outliers with dots. Note that the 13 subgroups had similar distributions of the Consortium lung disease phenotype.

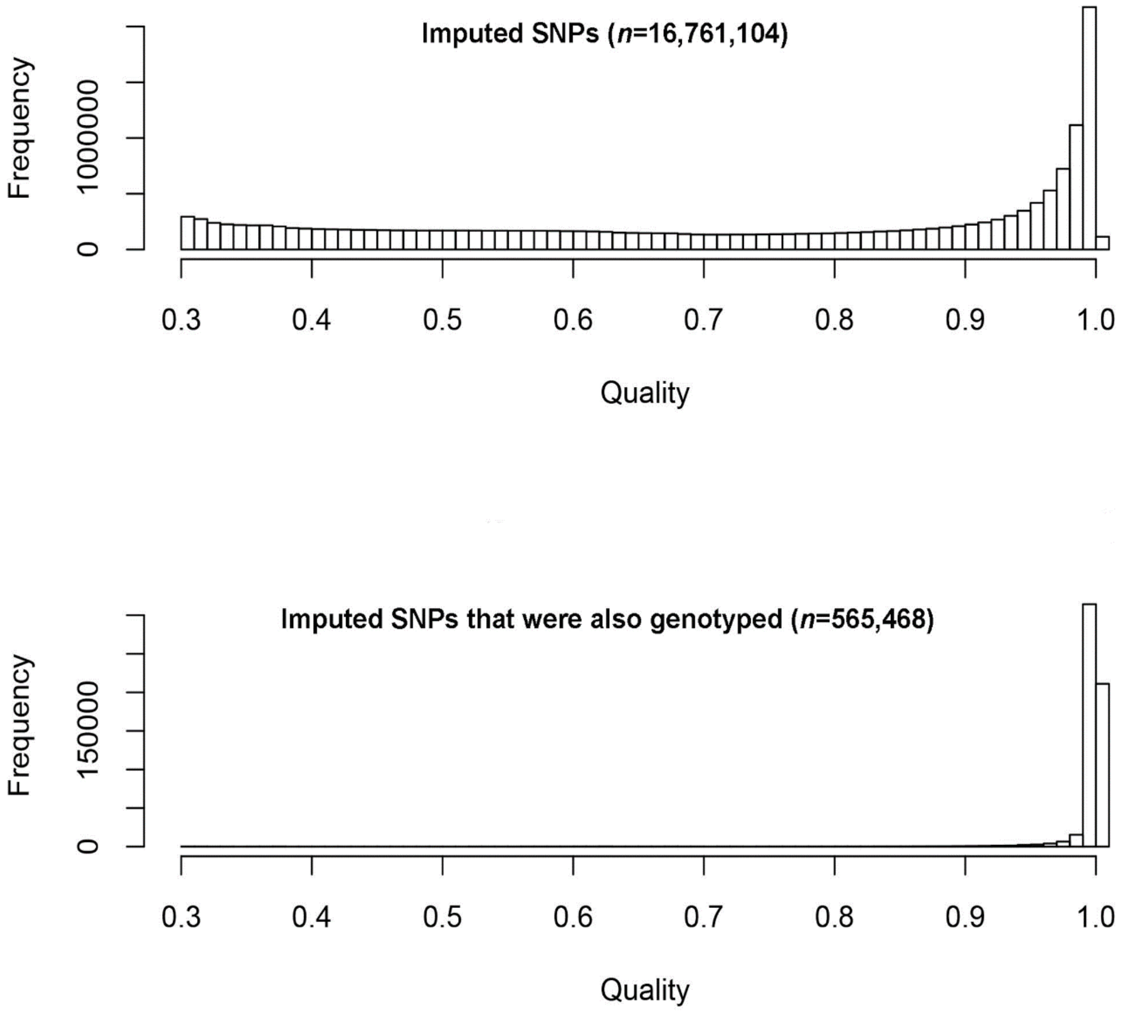

Supplementary Figure 2

Histogram for distribution of quality scores from imputation.

(**Top**) Histogram plot for the distribution of quality scores for SNPs imputed from genotyped SNPs on the 610 platform. The vast majority of imputed SNPs had quality scores near 1, indicating high quality. The other genotyping platforms had similar distributions of quality scores. Only ~50% of these imputed SNPs were used for analysis (8,520,458), as approximately half had minor allele frequencies < 0.005. (**Bottom**) Corresponding histogram plot for the subset of imputed SNPs that were also genotyped. As expected, those SNPs were imputed with even higher quality.

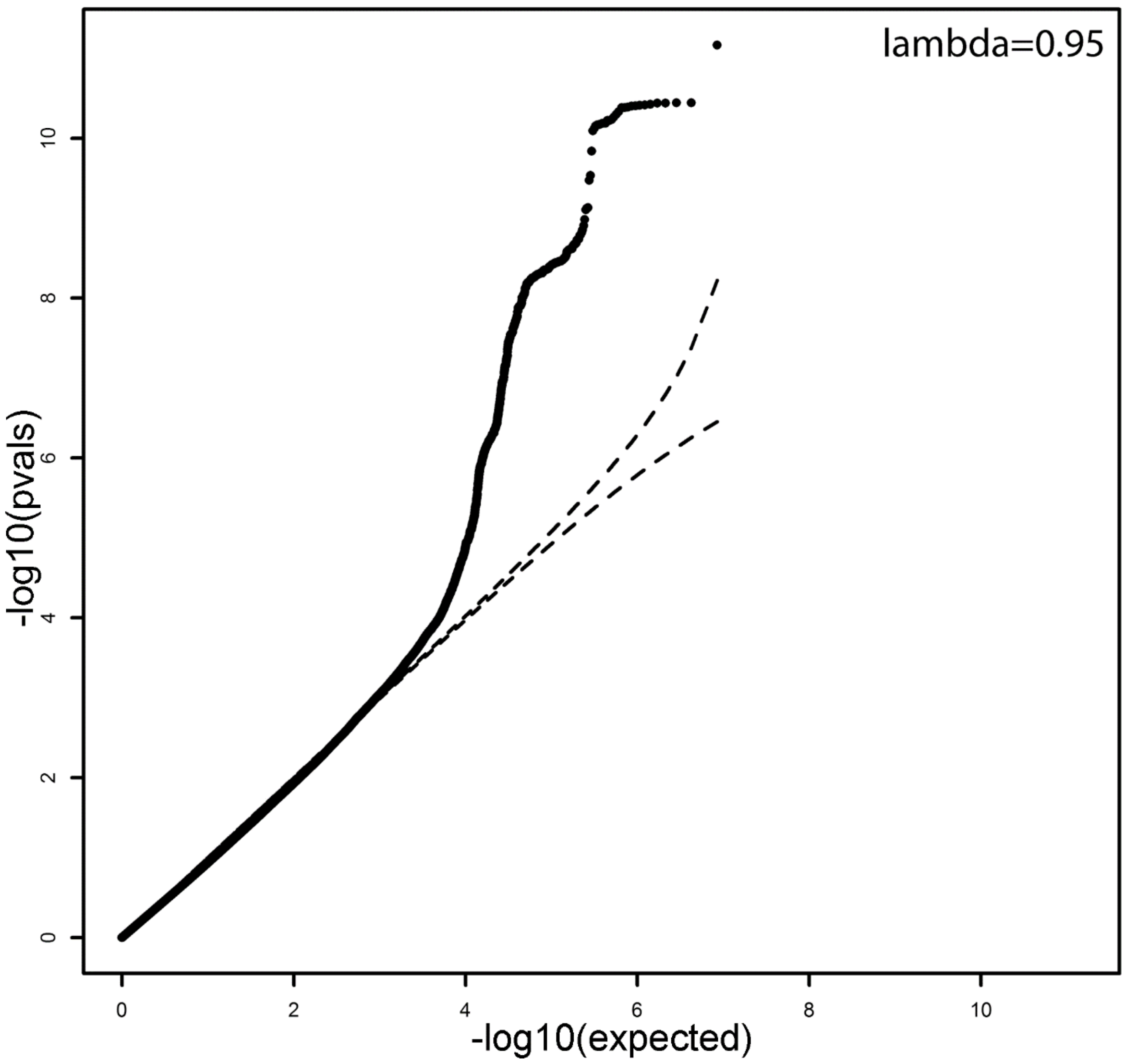

Supplementary Figure 3

Q-Q plot for the distribution of *P* values of imputed and genotyped SNPs using a random-effects model in the meta-analysis of the 13 GWAS1+2 subgroups for all individuals, with sex and principal components as covariates.

The genomic control value is lambda = 0.95, so the distribution of *P* values is not over-inflated. The genomic control value from the meta-analysis of genotyped SNPs common to all platforms was similar (lambda = 0.94; Q-Q plot not shown).

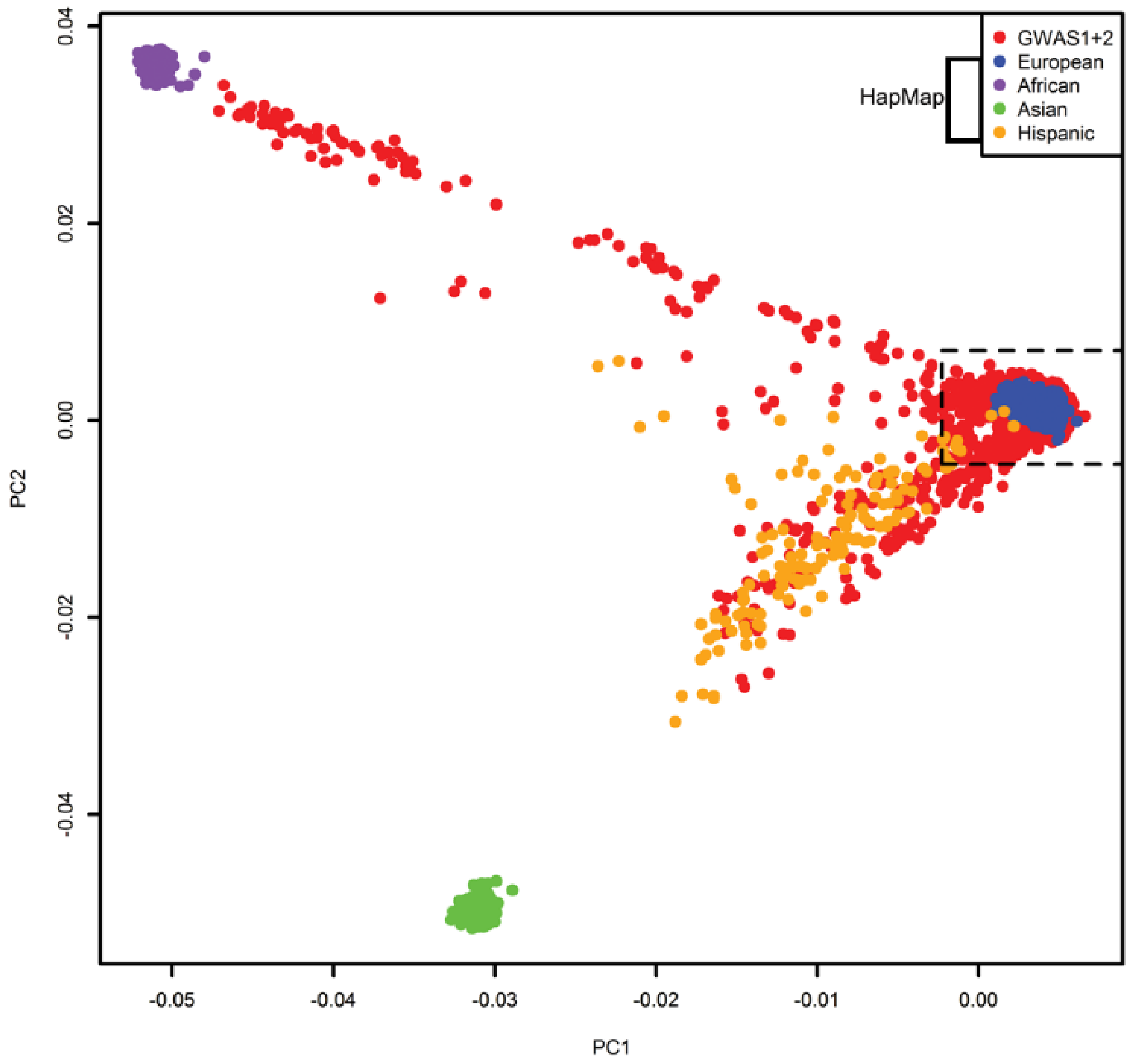

Supplementary Figure 4

Plot for the top two principal components from the joint analysis of GWAS1+2 individuals and HapMap3 individuals.

Individuals were judged to have European ancestry if principal components 1 and 2 fell within a mean ± 6SD black rectangle formed using principal components from the HapMap3 European dataset.

a

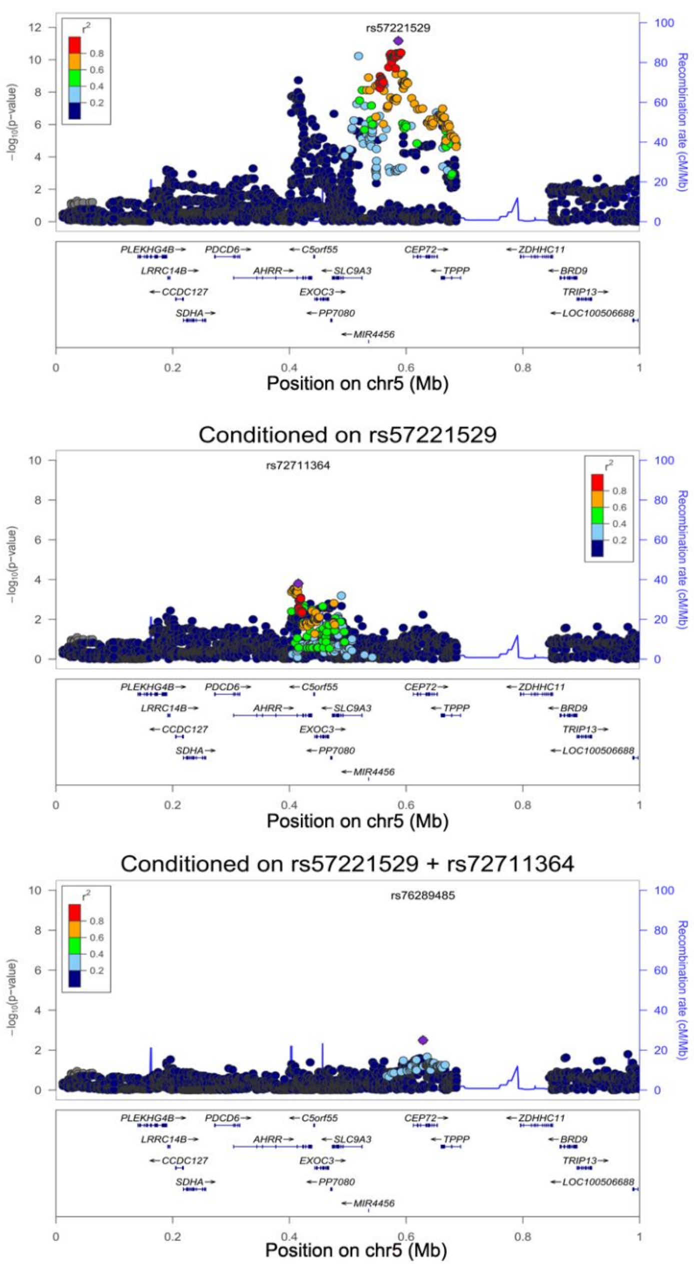

Supplementary Figure 5 (and next page)

Conditional analysis of the associations on chr5p15.3 (a) and 11p12-p13 (b).

b

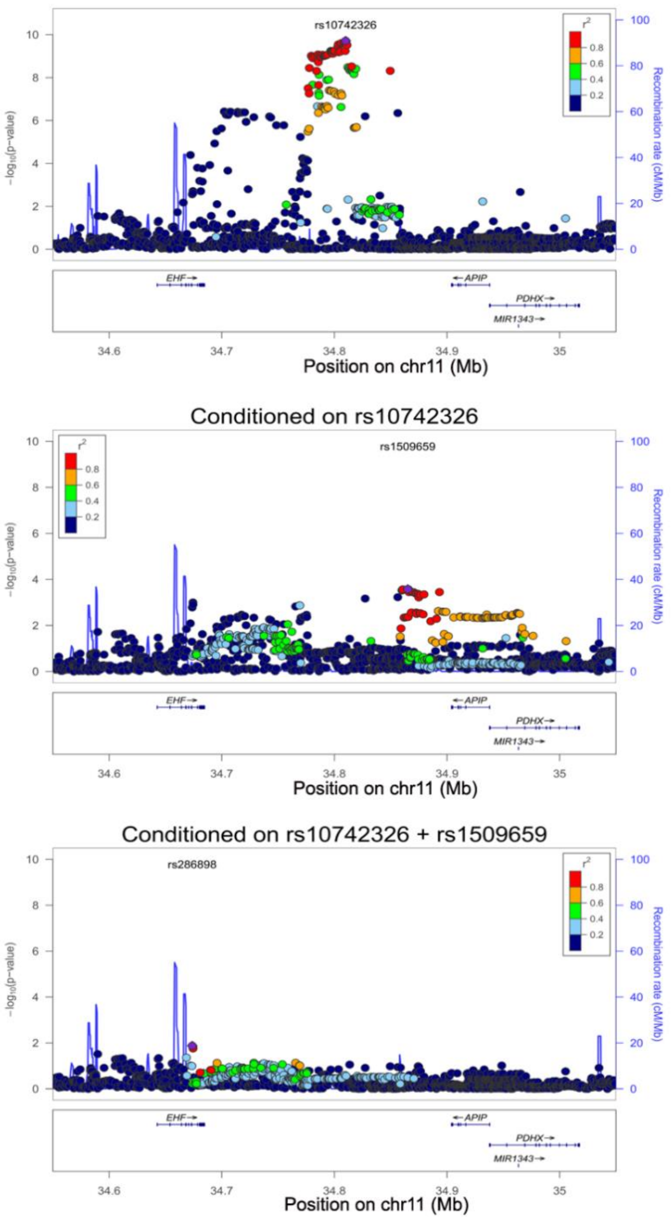

Supplementary Figure 5 (continued)

Conditional analysis of the associations on chr5p15.3 (a) and 11p12-p13 (b).

GEC software (http://statgenpro.psychiatry.hku.hk/gec/) was used to calculate the number of independent tests in each region. (**a**) The middle plot of this panel shows that after conditioning on the most significant SNP (rs57221529) at chr5p15.3 there are SNPs (near *AHRR*) that meet a regional Bonferroni significance threshold. (**b**) The middle plot of this panel shows that after conditioning on the most significant SNP (rs10742326) at chr11p12-p13 that there are SNPs (near/over *APIP*) that meet a regional Bonferroni significance threshold. (**a,b**) The bottom plots show that after conditioning on both SNPs (from the top and middle plots), that neither region has any significant association.

Supplementary Table 1. Associations for highest ranked SNP in each significant region by GWAS1 and/or GWAS2 for all patients and p.Phe508del homozygotes

Gene	Analysis	Effects	Id	Base pair	Minor allele	Minor allele frequency	Coefficient	*P* value	*P* value, Europeans	*P* value, Males	*P* value, Females
MUC4/ MUC20 (chr3)	GWAS1&2	Random	rs863582	195,478,694	G	0.33	0.11	1.39×10^{-07}	7.03×10^{-10}	6.84×10^{-07}	2.98×10^{-05}
	All	Fixed	rs3103933 *	195,485,440	A	0.37	0.12	3.27×10^{-11}	8.11×10^{-11}	1.52×10^{-07}	1.77×10^{-05}
	GWAS1&2	Random	rs2688482	195,529,118	T	0.31	0.12	7.22×10^{-08}	3.40×10^{-07}	0.007	6.09×10^{-06}
	p.Phe508del/p.Phe508del	Fixed	rs2688482	195,529,118	T	0.31	0.12	7.22×10^{-08}	3.40×10^{-07}	0.001	6.09×10^{-06}
	GWAS2	Random	rs863582	195,478,694	G	0.33	0.11	0.001	7.36×10^{-04}	1.02×10^{-04}	0.015
	All	Fixed	rs3103933	195,485,440	A	0.37	0.12	1.28×10^{-06}	1.28×10^{-05}	1.27×10^{-04}	0.001
	GWAS2	Random	rs2688482	195,529,118	T	0.31	0.12	7.64×10^{-05}	3.70×10^{-04}	0.043	8.22×10^{-04}
	p.Phe508del/p.Phe508del	Fixed	rs2688482	195,529,118	T	0.31	0.12	7.64×10^{-05}	3.70×10^{-04}	0.015	8.22×10^{-04}
	GWAS1	Random	rs78151003	195,489,534	G	0.45	0.12	4.10×10^{-06}	7.48×10^{-07}	0.012	3.25×10^{-04}
	All	Fixed	rs78151003	195,489,534	G	0.45	0.12	4.10×10^{-06}	7.48×10^{-07}	0.003	3.25×10^{-04}
	GWAS1	Random	rs9871110	195,625,326	A	0.47	0.09	1.18×10^{-04}	8.26×10^{-05}	0.003	0.012
	p.Phe508del/p.Phe508del	Fixed	3:195522714:GGGA_	195,522,714	R	0.26	0.14	6.90×10^{-05}	2.90×10^{-05}	0.023	4.21×10^{-04}
SLC9A3 (chr5)	GWAS1&2	Random	rs57221529 *	586,624	G	0.19	-0.16	6.81×10^{-12}	1.00×10^{-11}	1.66×10^{-06}	8.51×10^{-07}
	All	Fixed	5:584767:G_GGT	584,767	I	0.18	-0.16	4.62×10^{-12}	4.19×10^{-12}	7.58×10^{-07}	1.87×10^{-06}
	GWAS1&2	Random	rs56302516	518,434	T	0.24	-0.17	4.16×10^{-09}	5.78×10^{-08}	2.57×10^{-05}	0.049
	p.Phe508del/p.Phe508del	Fixed	rs56302516	518,434	T	0.24	-0.17	4.16×10^{-09}	8.49×10^{-10}	2.57×10^{-05}	0.049
	GWAS2	Random	rs57221529	586,624	G	0.19	-0.16	5.33×10^{-07}	2.11×10^{-07}	2.05×10^{-05}	4.42×10^{-04}
	All	Fixed	5:584767:G_GGT	584,767	I	0.18	-0.16	3.77×10^{-09}	2.20×10^{-09}	9.75×10^{-06}	1.10×10^{-04}
	GWAS2	Random	rs7726839	618,586	G	0.23	-0.17	1.83×10^{-07}	2.51×10^{-08}	3.01×10^{-05}	0.002
	p.Phe508del/p.Phe508del	Fixed	rs55726539	526,807	A	0.26	-0.26	8.69×10^{-08}	9.44×10^{-09}	1.81×10^{-05}	1.57×10^{-10}
	GWAS1	Random	rs56302516	518,434	T	0.24	-0.17	3.89×10^{-07}	5.40×10^{-07}	2.40×10^{-04}	5.03×10^{-04}
	All	Fixed	rs182219748	443,344	C	0.37	-0.12	1.80×10^{-07}	2.62×10^{-07}	1.12×10^{-04}	5.87×10^{-04}
	GWAS1	Random	rs4271701	513,785	A	0.39	-0.15	1.12×10^{-05}	1.14×10^{-05}	0.003	0.014
	p.Phe508del/p.Phe508del	Fixed	rs4271701	513,785	A	0.39	-0.15	1.12×10^{-05}	1.14×10^{-05}	0.003	0.001
HLA-DRA (chr6)	GWAS1&2	Random	rs140348826	32,434,135	A	0.33	-0.10	6.29×10^{-07}	5.93×10^{-05}	0.011	2.24×10^{-05}
	All	Fixed	rs117230773 *	32,433,741	A	0.36	-0.13	1.71×10^{-09}	7.18×10^{-09}	1.06×10^{-04}	5.66×10^{-06}
	GWAS1&2	Random	rs643889	32,575,918	T	0.33	-0.10	4.96×10^{-05}	1.11×10^{-04}	0.124	0.006
	p.Phe508del/p.Phe508del	Fixed	rs117230773	32,433,741	A	0.36	-0.13	3.56×10^{-06}	7.00×10^{-06}	0.034	3.17×10^{-05}
	GWAS2	Random	rs114742324	32,657,351	C	0.31	-0.10	3.87×10^{-04}	8.26×10^{-04}	0.012	0.192
	All	Fixed	rs114742324	32,657,351	C	0.31	-0.10	3.87×10^{-04}	8.26×10^{-04}	3.90×10^{-04}	0.104
	GWAS2	Random	rs116476006	32,375,431	C	0.09	-0.16	0.003	0.010	0.087	0.012
	p.Phe508del/p.Phe508del	Fixed	rs116476006	32,375,431	C	0.09	-0.16	0.003	0.010	0.087	0.012
	GWAS1	Random	rs117230773	32,433,741	A	0.36	-0.13	2.02×10^{-09}	1.81×10^{-08}	1.02×10^{-04}	8.66×10^{-06}
	All	Fixed	rs117230773	32,433,741	A	0.36	-0.13	2.02×10^{-09}	1.81×10^{-08}	1.02×10^{-04}	8.66×10^{-06}
	GWAS1	Random	rs117230773	32,433,741	A	0.36	-0.13	5.41×10^{-07}	1.05×10^{-06}	0.013	1.92×10^{-05}
	p.Phe508del/p.Phe508del	Fixed	rs114656294	32,433,276	G	0.50	0.15	2.60×10^{-07}	8.29×10^{-07}	0.002	3.05×10^{-05}
APIP/EHF (chr11)	GWAS1&2	Random	rs7112043	34,789,386	T	0.41	0.09	3.74×10^{-09}	6.06×10^{-08}	3.31×10^{-06}	0.003
	All	Fixed	rs546131	34,851,760	G	0.36	-0.09	3.46×10^{-09}	7.03×10^{-09}	1.37×10^{-05}	2.03×10^{-04}
	GWAS1&2	Random	rs10742326 *	34,810,010	A	0.42	0.12	1.92×10^{-10}	1.64×10^{-08}	2.21×10^{-07}	1.71×10^{-05}
	p.Phe508del/p.Phe508del	Fixed	rs7929679	34,805,849	G	0.50	0.11	1.91×10^{-10}	1.32×10^{-09}	1.49×10^{-07}	1.39×10^{-05}
	GWAS2	Random	11:34797572:GTTAT	34,797,572	D	0.42	0.07	7.14×10^{-04}	0.002	0.003	0.067
	All	Fixed	11:34797572:GTTAT	34,797,572	D	0.42	0.07	7.14×10^{-04}	0.002	0.003	0.039

	GWAS2	Random	11:34783993:TC_T	34,783,993	D	0.40	0.08	0.003	0.024	0.022	0.006
	p.Phe508del/p.Phe508del	Fixed	11:34783993:TC_T	34,783,993	D	0.40	0.08	0.003	0.016	0.022	0.005
	GWAS1	Random	rs523246	34,845,011	G	0.28	-0.13	6.05×10^{-09}	8.71×10^{-08}	3.76×10^{-04}	2.97×10^{-04}
	All	Fixed	rs508062	34,843,820	C	0.28	-0.13	6.01×10^{-09}	7.71×10^{-09}	3.67×10^{-04}	1.89×10^{-06}
	GWAS1	Random	rs11032874	34,806,607	G	0.50	0.15	2.32×10^{-10}	8.64×10^{-10}	6.75×10^{-04}	5.91×10^{-05}
	p.Phe508del/p.Phe508del	Fixed	rs11032874	34,806,607	G	0.50	0.15	2.32×10^{-10}	7.18×10^{-10}	1.12×10^{-06}	5.91×10^{-05}
AGTR2/ SLC6A14 (chrX)	GWAS1&2	Random	rs5952223 *	115,386,565	T	0.28	0.08	1.83×10^{-09}	1.25×10^{-09}	3.64×10^{-04}	2.84×10^{-05}
	All	Fixed	rs7879546	115,348,275	C	0.48	0.07	1.11×10^{-09}	4.55×10^{-10}	5.49×10^{-06}	1.02×10^{-05}
	GWAS1&2	Random	rs5952123	115,418,444	C	0.36	0.10	6.64×10^{-08}	1.44×10^{-07}	2.79×10^{-04}	0.006
	p.Phe508del/p.Phe508del	Fixed	rs5952223	115,386,565	T	0.28	0.08	3.30×10^{-08}	4.63×10^{-08}	9.49×10^{-06}	8.02×10^{-04}
	GWAS2	Random	rs5952120	115,401,529	C	0.27	0.09	2.29×10^{-06}	5.17×10^{-06}	1.86×10^{-05}	0.054
	All	Fixed	rs5952218	115,382,260	T	0.23	0.10	1.86×10^{-06}	1.32×10^{-06}	5.26×10^{-05}	0.027
	GWAS2	Random	rs5952123	115,418,444	C	0.36	0.10	1.65×10^{-05}	3.51×10^{-05}	0.003	0.159
	p.Phe508del/p.Phe508del	Fixed	rs145287377	115,387,123	C	0.21	0.14	2.48×10^{-06}	2.64×10^{-06}	2.00×10^{-05}	0.110
	GWAS1	Random	rs5952140	115,464,983	C	0.49	-0.08	1.51×10^{-06}	6.61×10^{-07}	7.21×10^{-04}	5.47×10^{-05}
	All	Fixed	rs12559259	115,348,321	G	0.45	0.10	1.66×10^{-07}	4.36×10^{-08}	0.004	8.03×10^{-08}
	GWAS1	Random	rs5952140	115,464,983	C	0.49	-0.08	9.40×10^{-07}	1.52×10^{-06}	2.03×10^{-04}	3.52×10^{-04}
	p.Phe508del/p.Phe508del	Fixed	rs12559259	115,348,321	G	0.45	0.10	2.23×10^{-07}	2.81×10^{-07}	0.001	1.38×10^{-05}

*Asterisks indicate SNPs of maximum significance between random- and fixed-effects meta-analysis and between all CFTR genotypes and p.Phe508del homozygotes P-values are derived from a meta-analysis using inverse variance-based weighting.

Supplementary Table 2. Replication-based analysis

Chr	SNP	*P* value, North American[a]	*P* value, French[a]
3	rs3103933	3.6×10^{-8}	8.7×10^{-5}
5	rs57221529	2.7×10^{-10}	0.003
6	rs116003090	4.1×10^{-8}	0.054[b]
11	rs10742326	9.3×10^{-9}	0.003
X	rs5952223	9.8×10^{-7}	1.8×10^{-4}

[a]Meta-analysis *P* values based on a fixed effects model, for the 11 North American and two French subgroups. [b]*P* values ranged from 0.030 to 0.054 for the six imputed SNPs that exceeded genome-wide significance in the meta-analysis.

Supplementary Table 3. eQTLs in lung, whole blood, LCLs and monocytes

A. GTEx: eQTLs for top-ranked GWAS SNP at each locus[a]

Chr	SNP	transcript	Lung eQTL *P* value	Blood eQTL *P* value
3	rs3103933	*MUC20*	0.001	3.6×10^{-4}
		AC0695133	0.005	0.001
		SDHAP2	0.012	0.018
		MUC4	0.498	N/A
5	rs57221529	*EXOC3*	0.017	0.002
		CTD-2228K2.7	0.022	0.042
		SLC9A3	0.179	0.054
		TPPP	0.236	0.017
6	rs116003090	*HLA-DRB1*	2.4×10^{-8}	8.1×10^{-10}
		HLA-DRB5	0.005	0.043
		HLA-DRA	0.040	0.29
		BTNL2	0.026	0.49
11	rs10742326	*APIP*	2.7×10^{-6}	0.02
		EHF	0.19	0.07
X			N/A	N/A

B. GTEx: Top-ranked eQTL at each locus[b]

Chr	SNP	r^2‡	transcript	Lung eQTL *P* value	Blood eQTL *P* value
3	rs2550262	0.67	*MUC20*	0.001	1.3×10^{-4}
			AC0695133	0.006	2.9×10^{-4}
			SDHAP2	0.008	0.020
			TNK	0.11	0.036
			MUC4	0.41	N/A
5	rs72703080	0.62	*CTD-2228K2.7*	6.4×10^{-4}	0.002
			SLC9A3	0.012	0.013
			EXOC3	0.023	0.011
			TPPP	0.589	0.032
6	rs146014754	0.79	*DRB1*	1.7×10^{-9}	3.7×10^{-11}
			DRB5	6.0×10^{-5}	7.9×10^{-4}
			DRA	0.032	0.39
			BTNL2	0.031	0.60
11	rs72920721	0.91	*APIP*	5.4×10^{-7}	0.006
			EHF	0.14	0.055
X				N/A	N/A

C. Other eQTL Databases: Top-ranked eQTL at each locus[c]

Chr	SNP	r^2‡	transcript	UChicago SCAN LCL eQTL *P* value	UNC seeQTL LCL or monocyte *Q* value
3	rs844518	0.63	*MUC20*	N/A	0.016
6	rs9268853	0.96	*HLA-DRB1*	5.0×10^{-7}	0.003
			HLA-DRB5	5.0×10^{-7}	5.2×10^{-24}
			HLA-DRA	N/A	2.1×10^{-4}
11	rs1588355	0.66	*APIP*	N/A	6.5×10^{-4}

eQTL P values from association testing in a linear regression model.

[a] eQTLs and genes with nominal *P* value < 0.05 in either lung and/or whole blood; data for two other genes (*MUC4* and *EHF*) are also shown, given biological plausibility for role in lung disease. N/A= not available. *P* values < 0.002 meet Bonferroni criteria (http://statgenpro.psychiatry.hku.hk/gec/) for significance at α = 0.05.

[b] Top-ranked regional eQTL *P* values (with ‡ r^2 > 0.6 with top-ranked GWAS SNPs from 3A, above).

[c] *P* values < 10^{-5} considered "significant" in the SCAN database, while seeQTL reports *Q* values for all cis-associations.

Supplementary Table 4. Association in GWAS1+2 for previously reported variants in candidate genes

Gene/locus	Chr	Variant rs#	Aliases	Previously reported phenotype(s)	Previously reported association *P* value	Previously reported number of subjects	*P* value, GWAS1+2 all (*n* = 6,365)	*P* value, GWAS1+2 p.Phe508del/ p.Phe508del (*n* = 4,139)
8.1AH	6	rs909253<br>rs1800629<br>rs1061581<br>rs1800625	+252 A>G<br>-308 G>A<br>1267 A>G<br>-429 T>C	FEV_1% pred	<0.04	404[a,1]	0.88<br>0.53<br>NA<br>0.1076	0.69<br>0.08<br>NA<br>0.3918
A1AT	14	rs17580<br>rs28929474	S allele<br>Z allele	FEV_1% pred	0.043	157[2]<br>215[3]<br>79[4]	0.34<br>0.76	0.36<br>0.83
ABCC1	16	rs504348	4741 C>G	Age at which FEV_1 <60%	<0.05	203[5]	0.64	0.97
ACE	17	none	indel	Age at which FEV_1 <50%	0.03	261[6]	Not tested	Not tested
ADBR2	5	rs1042713	Arg16Gly	FEV_1% pred<br>FVC<br>Flows at lower lung volumes<br>Longitudinal FEV_1	<0.05<br><0.05<br><0.01<br><0.01	126[7]	0.42	0.63
AGER	6	rs1800625	-429T>C	FEV_1<br>CF-specific percentile z-score<br>KNoRMA	0.02<br>0.03<br>0.03	967 [a,8]	0.11	0.39
CEACAM3	19	rs6508999 to<br>rs10414823		Disease severity	0.0469[b]	37 nuclear families[9]	0.20[c]<br>0.19[d]	0.51[c]<br>0.51[d]
CEACAM6	19	rs1549960 to<br>rs11548735		Disease severity	0.0106[b]	37 nuclear families[9]	0.31[c]<br>0.22[d]	0.23[c]<br>0.19[d]
EDNRA	4	rs5335	6672 G>C	Longitudinal FEV_1	0.002	1,577[10]	0.13	0.19
GCLC	6	none	$(GAG)_n$	FEV_1% pred	0.097<br>0.001 (mild)<br>0.533 (severe)	440[11]	Not tested	Not tested
GSTM1	1	none	GSTM1*0/<br>GSTM1*0	FEV_1% pred<br>Chrispin-Norman score<br>Shwachman score	0.16<br>0.02<br>0.04	53[12]<br>194[13]<br>60[14]	Not tested	Not tested
GSTM3	1	rs1799735	GSTM3*A<br>GSTM3*B	FEV_1<br>FVC	0.01<br>0.002	146[15]	NA	NA
HFE	6	rs1800562<br>and/or<br>rs1799945	C282Y<br>and/or<br>H63D	FEV_1% pred<br>FVC% pred<br>Annual change in FEV_1% pred<br>Annual change in FVC% pred	0.03<br>0.02<br>0.003<br>0.001	82[16]	0.29<br><br>0.06	0.20<br><br>0.78
HMOX1	22	rs2071749	11354 A>G	FEV_1% pred	0.01	755 [a,17]	0.12	0.15
IFRD1	7	rs7817	57460 C>T	Cross-sectional measures of lung function<br>Longitudinal measures of lung function	0.004<br>0.016	320[18]	0.94	0.32

				FEV_1% pred (adjusted)	No Association	1,978[1]		
		rs3807213	47556 G>T	Longitudinal measures of lung function	0.080		0.95	0.45
		rs6968084	38923 C>T	Cross-sectional measures of lung function	0.082		0.81	0.84
IL1b	2	rs1143634<br>rs1143639		Longitudinal FEV_1	0.120<br>0.057	808 [a,19]<br>126 families [a,19]	0.33<br>0.34	0.23<br>0.23
IL8	4	rs2227306<br>rs2227307<br>rs2227543<br>rs4073	+781C>T<br>+396T>G<br><br>-251 A>T	Longitudinal FEV_1	0.19<br>0.04<br>0.06<br>0.07	737/385 [a,20]<br>727/385 [a,20]<br>732/385 [a,20]<br>733/385 [a,20]	0.06<br>0.11<br>0.06<br>0.06	0.29<br>0.23<br>0.28<br>0.12
MBL2	10	rs1800450<br>rs1800451	X1 – B (A>G)<br>X1 – C (A>G)	FEV_1%<br>FVC%	0.003<br>0.03	149[21]<br>164[22]<br>179[23]<br>298[24]<br>47[25]<br>135[26]<br>254[27]<br>105[28]	0.79<br>0.95	0.34<br>0.66
NOS1		none	$(GT)_{18-36}$	Longitudinal FEV_1	0.025	59[29]<br>40[30]	Not tested	Not tested
NOS3	7	rs1799983	894 G>T	FEV_1	0.08 (in females)	70[31]	0.88	0.84
PPP2R1A	19	rs2162779	c.*465T>A	Functional residual capacity	0.0033	95[32]	0.91	0.92
PPP2R4	9	rs3118625	c.-185A>C	FEV_1<br>Lung clearance index	0.0048<br>0.0059	95[32]	1.00	0.61
SERPINA3	14	rs4934	T-15A	FEV_1% pred	0.04	157[33]	0.45	0.53
SFTPA1	10	none	$6A^3$ & $6A^3/1A^1$ haplotype	FEV_1% pred	0.01	135[26]	Not tested	Not tested
SFTPA2	10	none	$1A^1$ & $6A^3/1A^1$ haplotype	FEV_1% pred	0.009<br>0.13	135[26]	Not tested	Not tested
SLC6A14	23	rs5905283<br>rs3788766		Longitudinal FEV_1	0.004<br>0.0002	815 [a,34]	0.23<br>0.54	0.59<br>0.34
SLC9A3	5	rs4957061<br>rs17563161	521096 C>T	Longitudinal FEV_1<br>KNoRMA	0.05<br>1.5×10^{-6}	752 [a,35]<br>815 [a,34]	5.69×10^{-6}<br>1.17×10^{-4}	7.93×10^{-6}<br>3.58×10^{-3}
SNAP23	15	rs9302112	c.267-9T>C	FEF_{50}<br>Functional residual capacity<br>Volume of trapped gas	0.0088<br>0.011<br>0.0043	95 [a,32]	0.20	0.61
TGFB1	19	rs1800470<br><br>rs1800469	codon 10<br>C29T<br>C-509T	Age at which FEV_1 <50%<br>Age at which FVC <70%<br>Impairment of lung function (FEV_1)	<0.02<br><0.005<br>0.006	171[36]<br>808 [a,37]<br>261[6]<br>808 [a,37]<br>498 [a,37]<br>329 [a,38]<br>105[28]<br>472 [a,39]	0.78<br><br>0.47	0.47<br><br>0.54

TNFA	6	rs1800629	G-308A<br>+691G ins/del	Mean FEV_1% pred	0.02<br>0.008	53[12]<br>180[40]	0.53	0.08

Variants previously reported in candidate gene studies (as of May/June 2014) as associating with some aspect of pulmonary function in CF patients (Phenotype Tested) are listed here with their rs#, other names of the variants (aliases), the reported p-values, the number of subjects tested to achieve that p-value and the publication reporting the association. The right-hand columns give the meta-analysis *P* value calculated using inverse variance-based weighting for those variants in this study, both for all subjects enrolled and for p.Phe508del homozygotes. The current study evaluated single nucleotide polymorphism only, so other types of variants, such as insertions or deletions (indels) or variable number of repeats were not tested. [a]These cohorts are not independent of the current study. [b]*P* value reflects the association of the haplotype to the lung disease. [c]*P* value reflects the association of the first SNP in the previously reported haplotype range to the GWAS1+2 phenotype. [d]*P* value reflects the association of the last SNP in the previously reported haplotype range to the GWAS1+2 phenotype.

SUPPLEMENTARY REFERENCES

1. Corvol, H. *et al.* Ancestral haplotype 8.1 and lung disease severity in European cystic fibrosis patients. *J Cyst Fibros* **11**, 63-7 (2012).
2. Mahadeva, R. *et al.* Alpha1-antitrypsin deficiency alleles and the Taq-I G-->A allele in cystic fibrosis lung disease. *European Respiratory Journal* **11**, 873-879 (1998).
3. Doring, G., Krogh-Johansen, H., Weidinger, S. & Hoiby, N. Allotypes of alpha 1-antitrypsin in patients with cystic fibrosis, homozygous and heterozygous for deltaF508. *Pediatric Pulmonology* **18**, 3-7 (1994).
4. Mahadeva, R., Stewart, S., Bilton, D. & Lomas, D.A. Alpha-1 antitrypsin deficiency alleles and severe cystic fibrosis lung disease. *Thorax* **53**, 1022-1024 (1998).
5. Mafficini, A. *et al.* Impact of polymorphism of Multidrug Resistance-associated Protein 1 (ABCC1) gene on the severity of cystic fibrosis. *J Cyst Fibros* **10**, 228-33 (2011).
6. Arkwright, P.D. *et al.* End-organ dysfunction in cystic fibrosis: association with angiotensin I converting enzyme and cytokine gene polymorphisms. *American Journal of Respiratory and Critical Care Medicine* **167**, 384-389 (2003).
7. Buscher, R. *et al.* beta2 adrenoceptor gene polymorphisms in cystic fibrosis lung disease. *Pharmacogenetics* **12** 347-346 (2002).
8. Beucher, J. *et al.* AGER -429T/C is associated with an increased lung disease severity in cystic fibrosis. *PLoS ONE* **7**, e41913 (2012).
9. Stanke, F. *et al.* Hierarchical fine mapping of the cystic fibrosis modifier locus on 19q13 identifies an association with two elements near the genes CEACAM3 and CEACAM6. *Hum Genet* **127**, 383-94 (2010).
10. Darrah, R. *et al.* EDNRA variants associate with smooth muscle mRNA levels, cell proliferation rates, and cystic fibrosis pulmonary disease severity. *Physiol.Genomics* **41**, 71-77 (2010).
11. McKone, E.F. *et al.* Variants in the glutamate-cysteine-ligase gene are associated with cystic fibrosis lung disease (e-pub ahead of print). *American Journal of Respiratory and Critical Care Medicine* **174**, 415-419 (2006).
12. Hull, J. & Thomson, A.H. Contribution of genetic factors other than CFTR to disease severity in cystic fibrosis. *Thorax* **53**, 1018-1021 (1998).
13. Baranov, V.S. *et al.* Proportion of the GSTM1 0/0 genotype in some Slavic populations and its correlation with cystic fibrosis and some multifactorial diseases. *Human Genetics* **97**, 516-520 (1996).
14. Korytina, G.F., Iaibaeva, D.G. & Viktorova, T.V. [Polymorphism of glutathione-S-transferase M1 and P1 genes in patients with cystic fibrosis and chronic respiratory tract diseases]. *Genetika* **40**, 401-8 (2004).
15. Flamant, C. *et al.* Glutathione-S-transferase M1, M3, P1 and T1 polymorphisms and severity of lung disease in children with cystic fibrosis. *Pharmacogenetics* **14** 295-301 (2004).
16. Pratap, U., Quinn, S., Blizzard, L.B. & Reid, D.W. Population-based study of cystic fibrosis disease severity and haemochromatosis gene mutations. *Respirology* **15**, 141-9 (2010).
17. Park, J.E. *et al.* Cystic fibrosis modifier genes related to Pseudomonas aeruginosa infection. *Genes Immun* **12**, 370-7 (2011).
18. Gu, Y. *et al.* Identification of IFRD1 as a modifier gene for cystic fibrosis lung disease. *Nature* **458**, 1039-1042 (2009).
19. Levy, H. *et al.* IL1B polymorphisms modulate cystic fibrosis lung disease. *Pediatric Pulmonology* **44**, 580-593 (2009).

20. Hillian, A.D. *et al.* Modulation of cystic fibrosis lung disease by variants in interleukin-8. *Genes and Immunity* **9**, 501-508 (2008).
21. Garred, P. *et al.* Association of mannose-binding lectin gene heterogeneity with severity of lung disease and survival in cystic fibrosis [see comments]. *Journal of Clinical Investigation* **104**, 431-437 (1999).
22. Gabolde, M., Guilloud-Bataille, M., Feingold, J. & Besmond, C. Association of variant alleles of mannose binding lectin with severity of pulmonary disease in cystic fibrosis: cohort study. *British Medical Journal* **319**, 1166-1167 (1999).
23. Yarden, J. *et al.* Polymorphisms in the mannose binding lectin gene affect the cystic fibrosis pulmonary phenotype. *Journal of Medical Genetics* **41**, 629-633 (2004).
24. Davies, J.C., Turner, M.W., Klein, N. & the London, M.B.L.C.F.S.G. Impaired pulmonary status in cystic fibrosis adults with two mutated MBL-2 alleles. *European Respiratory Journal* **24** 298-804 (2004).
25. Trevisiol, C. *et al.* MBL2 polymorphisms screening in a regional Italian CF Center. *J Cyst.Fibros.* **4**, 189-191 (2005).
26. Choi, E.H., Ehrmantraut, M., Foster, C.B., Moss, J. & Chanock, S.J. Association of common haplotypes of surfactant protein A1 and A2 (SFTPA1 and SFTPA2) genes with severity of lung disease in cystic fibrosis. *Pediatric Pulmonology* **41**, 255-262 (2006).
27. Buranawuti, K. *et al.* Variants in mannose-binding lectin and tumour necrosis factor alpha affect survival in cystic fibrosis. *J Med Genet* **44**, 209-14 (2007).
28. Faria, E.J. *et al.* Association of MBL2, TGF-beta1 and CD14 gene polymorphisms with lung disease severity in cystic fibrosis. *J Bras.Pneumol.* **35**, 334-342 (2009).
29. Texereau, J. *et al.* Nitric oxide synthase 1 as a potential modifier gene of decline in lung function in patients with cystic fibrosis. *Thorax* **59**, 156-8 (2004).
30. Grasemann, H. *et al.* Nasal nitric oxide levels in cystic fibrosis patients are associated with a neuronal NO synthase (NOS1) gene polymorphism. *Nitric Oxide* **6**, 236-41 (2002).
31. Grasemann, H. *et al.* Endothelial nitric oxide synthase variants in cystic fibrosis lung disease. *Am J Respir Crit Care Med* **167**, 390-4 (2003).
32. Gisler, F.M., von Kanel, T., Kraemer, R., Schaller, A. & Gallati, S. Identification of SNPs in the cystic fibrosis interactome influencing pulmonary progression in cystic fibrosis. *Eur J Hum Genet* **21**, 397-403 (2013).
33. Mahadeva, R. *et al.* Association of alpha(1)-antichymotrypsin deficiency with milder lung disease in patients with cystic fibrosis. *Thorax* **56**, 53-58 (2001).
34. Li, W. *et al.* Unraveling the complex genetic model for cystic fibrosis: pleiotropic effects of modifier genes on early cystic fibrosis-related morbidities. *Human Genetics* **133**, 151-161 (2014).
35. Dorfman, R. *et al.* Modulatory effect of the SLC9A3 gene on susceptibility to infections and pulmonary function in children with cystic fibrosis. *Pediatric Pulmonology* **46**, 385-392 (2011).
36. Arkwright, P.D. *et al.* TGF-beta(1) genotype and accelerated decline in lung function of patients with cystic fibrosis. *Thorax* **55**, 459-462 (2000).
37. Drumm, M.L. *et al.* Gene modifiers of lung disease in cystic fibrosis. *New England Journal of Medicine* **353**, 1443-1453 (2005).
38. Corvol, H. *et al.* Genetic variations in inflammatory mediators influence lung disease progression in cystic fibrosis. *Pediatric Pulmonology* **43**, 1224-1232 (2008).
39. Bremer, L.A. *et al.* Interaction between a novel TGFB1 haplotype and CFTR genotype is associated with improved lung function in cystic fibrosis. *Human Molecular Genetics* **17**, 2228-2237 (2008).
40. Yarden, J. *et al.* Association of tumour necrosis factor alpha variants with the CF pulmonary phenotype. *Thorax* **60**, 320-325 (2005).

Contributing French cystic fibrosis centers and principal investigators:

M. Abely, American Memorial Hospital, Reims, France; L. Bassinet, Centre Hospitalier Intercommunal de Créteil, Créteil, France; C. Belleguic, Hôpital Pontchaillou, Rennes, France; G. Bellon, Hôpital Femme Mère Enfant, Bron, France; K. Bessaci, American Memorial Hospital, Reims, France; A.S. Bonnel, Hôpital André Mignot, Le Chesnay, France; F. Bremont, Hôpital des Enfants de Toulouse, Toulouse, France; J. Brouard, Centre Hospitalier Universitaire de Caen, Caen, France; S. Bui, Hôpital des Enfants Groupe Pellegrin, Bordeaux, France; K. Campbell, Centre Hospitalier Universitaire de Caen, Caen, France; R. Chiron, Hôpital Arnaud de Villeneuve, Montpellier, France; R. Chumbi-Flores, Hôpital de la Tronche, Grenoble, France; J.C. Dalphin, CNRS-UFC, UMR 6249 Chrono-Environnement, Hôpital Jean Minjoz, Besançon, France; M.L. Dalphin, Centre Hospitalier Universitaire de Besançon, Besançon, France; V. David, Hôpital Mère-Enfant, Nantes, France; S. De Miranda, Hôpital Foch, Suresnes, France; J. Derelle, Hôpital d'Enfants, Vandoeuvre les Nancy, France; P. Domblides, Hôpital Haut Lévêque, Pessac, France; S. Dominique, Centre Hospitalier Universitaire Charles Nicolle, Rouen, France; J.C. Dubus, Hôpital d'Enfants de la Timone, Marseille, France; I. Durieu, UCBL1,Groupe Hospitalier Lyon Sud–Hospices Civils de Lyon, Pierre Bénite, France; S. Dury, Hôpital Maison Blanche, Reims, France; R. Epaud, Centre Hospitalier Intercommunal de Créteil, Créteil, France; A. Fanton, Hôpital d'Enfants du Bocage, Dijon, France; M. Fayon, Hôpital des Enfants Groupe Pellegrin, Bordeaux, France; E. Fleurence, Hôpital d'Enfants, Saint-Denis de la Réunion, France; P. Foucaud, Hôpital André Mignot, Le Chesnay, France; J.L. Ginies, Centre Hospitalier Universitaire d'Angers, Angers, France; B. Godbert, Hôpital de Brabois, Vandoeuvre les Nancy, France; D. Grenet, Hôpital Foch, Suresnes, France; M. Guillot, Centre Hospitalier Robert Bisson, Lisieux, France; A. Haloun, Hôpital Laënnec, Nantes, France ; M.C. Heraud, Centre Hospitalier Estaing, Clermont-Ferrand, France; B. Housset, Centre Hospitalier Intercommunal de Créteil, Créteil, France; A.C. Henriet, Hôpital Bretonneau, Tours, France ; D. Hubert, Hôpital Cochin, Paris, France; F. Huet, Hôpital d'Enfants du Bocage, Dijon, France; R. Kessler, Hôpital Civil, Strasbourg, France; A. Labbé, Centre Hospitalier Estaing, Clermont-Ferrand, France; M. Laurans, Centre Hospitalier Universitaire de Caen, Caen, France; M. Le Bourgeois, Hôpital Necker–Enfants Malades, Paris, France; P. Le Roux, Hôpital Jacques Monod, Montivilliers, France; C. Llerena, Hôpital de la Tronche, Grenoble, France; G.A. Loeuille, Centre Hospitalier de Dunkerque, Dunkerque, France; S. Marchand, Hôpital Clocheville, Tours, France ; C. Marguet, Centre Hospitalier Universitaire Charles Nicolle, Rouen, France; L. Melly, Hôpital Renée Sabran, Giens, France; V. Moisan-Petit, Centre Hospitalier Bretagne Atlantique, Vannes, France; G. Mousset, Centre Hospitalier Bretagne Atlantique, Vannes, France; A. Munck, Hôpital Robert Debré, Paris, France; M. Murris-Espin, Hôpital Larrey, Toulouse, France; R. Nove-Josserand, Groupe Hospitalier Lyon Sud–Hospices Civils de Lyon, Pierre Bénite, France; J.C. Pautard, Hôpital Nord, Amiens, France; I. Pin, INSERM, U823 Université Joseph Fourier, Hôpital de la Tronche, Grenoble, France; S. Pramil, Centre Hospitalier Universitaire Charles Nicolle, Rouen, France; A. Prevota, Hôpital Calmette, Lille, France; C. Rames, Hôpital Nord, Amiens, France; G. Rault, Centre de Perharidy, Roscoff, France; P. Reix, Hôpital Femme Mère Enfant, Bron, France; N. Remus, Centre Hospitalier Intercommunal de Créteil, Créteil, France; M. Renouil, Groupe Hospitalier Sud Réunion, Saint-Pierre de la Réunion, France; M. Reynaud-Gaubert, Hôpital Nord, Marseille, France; B. Richaud Thiriez, Hôpital Jean Minjoz, Besançon, France; M. Roussey, Université de Rennes 1, Hôpital Sud Annexe Pédiatrique, Rennes, France; I. Sermet-Gaudelus, Hôpital Necker–Enfants Malades, Paris, France; V. Storni, Centre Hospitalier Bretagne Atlantique, Vannes, France; N. Stremler, Hôpital d'Enfants de la Timone, Marseille, France; T. Urban, Centre Hospitalier Universitaire d'Angers, Angers, France; P. Vigneron, Centre Hospitalier Bretagne Sud, Lorient, France; B. Wallaert, Hôpital Calmette, Lille, France; L. Weiss, Hôpital de Hautepierre, Strasbourg, France ; N. Wizla, Hôpital Jeanne de Flandres, Lille, France.

Contributing North American cystic fibrosis centers and principal investigators:
S. Aaron, Ottawa General Hospital, Ottawa, Ontario, Canada; I. Abdulhamid, Children's Hospital of Michigan, Detroit, Michigan, USA; F. Accurso, University of Colorado Health Sciences Center, Aurora, Colorado, USA; J. Acton, Cincinnati Children's Hospital and Medical Center, Cincinnati, Ohio, USA; R. Ahrens, University of Iowa Hospitals & Clinics, Iowa City, Iowa, USA; G. Aljadeff, Lutheran General Children's Hospital, Park Ridge, Illinois, USA; C. Allard, Centre de Santé et de Services Sociaux de Chicoutimi, Chicoutimi, Quebec, Canada; R. Amaro, University of Texas at Tyler Health Center, Tyler, Texas, USA; R. Anbar, State University of New York (SUNY) Upstate Medical University, Syracuse, New York, USA; P. Anderson, University of Arkansas, Little Rock, Arkansas, USA; A. Atlas, Morristown Memorial Hospital, Morristown, New Jersey, USA; S. Bell, The Prince Charles Hospital, Brisbane, Queensland, Australia; M. Berdella, St. Vincent's Hospital & Medical Center, New York, New York, USA; Y. Berthiaume, Hôtel Dieu De Montréal, Montreal, Quebec, Canada; J. Biller, Children's Hospital of Wisconsin, Milwaukee, Wisconsin, USA; G. Bishop, Saint John Regional Hospital, Saint John, New Brunswick, Canada; C. Bjornson, Alberta Children's Hospital, Calgary, Alberta, Canada; P. Black, Children's Mercy Hospital, Kansas City, Missouri, USA; H. Black, Asthma & Allergy Specialists, Charlotte, North Carolina, USA; S. Blackman, Johns Hopkins Hospital, Baltimore, Maryland, USA; S. Boas, Children's Asthma Respiratory & Exercise Specialists, Glenview, Illinois, USA; M. Boland, Children's Hospital of Eastern Ontario, Ottawa, Ontario, Canada; D. Borowitz, Women & Children's Hospital of Buffalo, Buffalo, New York, USA; R. Boswell, University of Tennessee, Memphis, Tennessee, USA; J. Boucher, Centre Hospitalier Régional de Rimouski, Rimouski, Quebec, Canada; C.M. Bowman, Medical University of South Carolina, Charleston, South Carolina, USA; M. Boyle, Johns Hopkins Hospital, Baltimore, Maryland, USA; C. Brown, California Pacific Medical Center, San Francisco, California, USA; D. Brown, Pediatric Pulmonary Associates, Columbia, South Carolina, USA; N. Brown, University of Alberta Hospitals, Edmonton, Alberta, Canada; J. Brusky, University Hospital, Saskatoon, Saskatchewan, Canada; L.F. Caffey, University of New Mexico, Albuquerque, New Mexico, USA; C. Cannon, Children's Medical Center of Dallas, Dallas, Texas, USA; A. Cantin, Centre Hospitalier Universitaire de Sherbrooke, Fleurimont, Sherbrooke, Quebec, Canada; B. Chatfield, University of Utah, Salt Lake City, Utah, USA; S. Chesrown, University of Florida, Gainesville, Florida, USA; A. Chidekel, Alfred I. duPont Hospital for Children, Wilmington, Delaware, USA; M. Chilvers, British Columbia Children's Hospital, Vancouver, British Columbia, Canada; B. Chipps, Sutter Medical Center, Sacramento, California, USA; J.P. Clancy, University of Alabama at Birmingham, Birmingham, Alabama, and Cincinnati Children's Hospital, Cincinnati, Ohio, USA; R. Cohen, Kaiser Permanente, Portland, Oregon, USA; J. Colombo, University of Nebraska Medical Center, Omaha, Nebraska, USA; J. Cronin, Women & Children's Hospital of Buffalo, Buffalo, New York, USA; M. Cruz, St. Mary's Medical Center, West Palm Beach, Florida, USA; J. Cunningham, Cook Children's Medical Center, Fort Worth, Texas, USA; G. Cutting, Johns Hopkins Hospital, Baltimore, Maryland, USA; G. Davidson, British Columbia Children's Hospital, Vancouver, British Columbia, Canada; D. Davies, Michigan State University, East Lansing, Michigan, USA; L. Davies, University of New Mexico, Albuquerque, New Mexico, USA; D. Debray, Centre Hospitalier Universitaire de Bicêtre, Bicêtre, France; J. DeCelie-Germana, Schneider Children's Hospital, New Hyde Park, New York, USA; A. Devenny, Royal Hospital for Sick Children, Glasgow, Scotland, UK; E. DiMango, Columbia University Medical Center, New York, New York, USA; D. Doornbos, Via-Christi St. Francis, Wichita, Kansas, USA; H. Dorkin, Children's Hospital Boston, Boston, Massachusetts, USA; M. Dovey, Children's Hospital Boston, Boston, Massachusetts, USA; A. Dozor, New York Medical College–Westchester Medical Center, Valhalla, New York, USA; J. Dunitz, University of Minnesota, Minneapolis, Minnesota, USA; M. Egan, Yale University School of Medicine, New Haven, Connecticut, USA; J. Eichner, Great Falls Clinic, Great Falls, Montana, USA; J. Farrell, Janeway Child Health Centre, St. John's, Newfoundland and

Labrador, Canada; T. Ferkol, St. Louis Children's Hospital, St. Louis, Missouri, USA; S. Fiel, Morristown Memorial Hospital, Morristown, New Jersey, USA; P. Flume, Medical University of South Carolina, Charleston, South Carolina, USA; M. Franco, Miami Children's Hospital, Miami, Florida, USA; A. Freitag, Hamilton Health Sciences Corporation, Hamilton, Ontario, Canada; D. Froh, University of Virginia Health System, Charlottesville, Virginia, USA; N. Garey, Saint John Regional Hospital, Saint John, New Brunswick, Canada; D. Geller, Nemours Children's Clinic, Orlando, Florida, USA; W. Gershan, Children's Hospital of Wisconsin, Milwaukee, Wisconsin, USA; R. Gibson, Children's Hospital & Regional Medical Center, Seattle, Washington, USA; R. Giusti, Long Island College Hospital, Brooklyn, New York, USA; J. Gjevre, Royal University Hospital, Saskatoon, Saskatchewan, Canada; S. Goldfinger, St. Mary's Medical Center, West Palm Beach, Florida, USA; M. Gondor, University of South Florida, St. Petersburg, Florida, USA; G. Gong, Phoenix Children's Hospital, Phoenix, Arizona, USA; S. Goulet, Centre Hospitalier Régional de Rimouski, Rimouski, Quebec, Canada; D. Green, Duke University, Durham, North Carolina, USA; M. Guill, Medical College of Georgia, Augusta, Georgia, USA; H. Gutierrez, University of Alabama at Birmingham, Birmingham, Alabama, USA; A. Hadeh, Drexel University College of Medicine, Philadelphia, Pennsylvania, USA; D. Hale, Baylor College of Medicine, Houston, Texas, USA; K. Hardy, Children's Hospital, Oakland, California, USA; T. Hazinski, Vanderbilt University Medical Center, Nashville, Tennessee, USA; K. Henderson, Janeway Health and Rehabilitation Centre, St. John's, Newfoundland and Labrador, Canada; P. Hiatt, Texas Children's Hospital, Houston, Texas, USA; D. Hicks, Children's Hospital of Orange County, Orange, California, USA; J. Hoag, Drexel University College of Medicine, Philadelphia, Pennsylvania, USA; B. Holmes, Regina General Hospital, Regina, Saskatchewan, Canada; D. Holsclaw, University of Pennsylvania, Philadelphia, Pennsylvania, USA; P. Holzwarth, St. Vincent Hospital, Green Bay, Wisconsin, USA; R. Honicky, Michigan State University, East Lansing, Michigan, USA; M. Howenstine, Riley Hospital for Children, Indianapolis, Indiana, USA; D. Hughes, IWK Health Centre, Halifax, Nova Scotia, Canada; M. Jackson, Grand River Hospital, Kitchener, Ontario, Canada; P. James, Lutheran Hospital, Fort Wayne, Indiana, USA; A. Jenneret, Hôtel Dieu de Montréal, Montreal, Quebec, Canada; P. Joseph, University of Cincinnati, Cincinnati, Ohio, USA; J. Kanga, University of Kentucky, Lexington, Kentucky, USA; M. Katz, Baylor College of Medicine, Houston, Texas, USA; S. Kent, Victoria General Hospital, Victoria, British Columbia, Canada; W. Kepron, Health Sciences Centre, Winnipeg, Manitoba, Canada; E. Kerem, Hadassah Medical Center, Jerusalem, Israel; D. Kissner, Wayne State University, Detroit, Michigan, USA; M. Knowles, University of North Carolina at Chapel Hill, Chapel Hill, North Carolina, USA; P. Konig, University of Missouri, Columbia, Missouri, USA; M. Konstan, Case Western Reserve University, Cleveland, Ohio, USA; T. Kovesi, Children's Hospital of Eastern Ontario, Ottawa, Ontario, Canada; J. Kramer, Oklahoma Cystic Fibrosis Center, Tulsa, Oklahoma, USA; N. Kraynack, Children's Hospital Medical Center of Akron, Akron, Ohio, USA; V. Kumar, Hôpital Régional de Sudbury Regional Hospital, Sudbury, Ontario, Canada; F. Lacaille, Hôpital Necker–Enfants Malades, Paris, France; T. Lahiri, Fletcher Allen Health Care, Burlington, Vermont, USA; C. Landon, Pediatric Diagnostic Center, Ventura, California, USA; L. Lands, Montréal Children's Hospital, Montreal, Quebec, Canada; A. Lapey, Massachusetts General Hospital, Boston, Massachusetts, USA; C. Lapin, Connecticut Children's Medical Center, Hartford, Connecticut, USA; M. Larj, Wake Forest University Baptist Medical Center, Winston-Salem, North Carolina, USA; A. Lavoie, Hôtel Dieu de Montréal, Montreal, Quebec, Canada; J. Ledbetter, TC Thompson Children's Hospital, Chattanooga, Tennessee, USA; R. Lee, Naval Medical Center, Portsmouth, Virginia, USA; M. Leigh, University of North Carolina at Chapel Hill, Chapel Hill, North Carolina, USA; L. Lester, University of Chicago Children's Hospital and Rush University Medical Center, Chicago, Illinois, USA; T. Lever, Eastern Maine Medical Center, Bangor, Maine, USA; H. Levy, Children's Hospital Boston, Boston, Massachusetts, and Medical College of Wisconsin, Milwaukee, Wisconsin, USA; A. Lieberthal,

Kaiser Permanente Southern California, Panorama City, California, USA; T. Liou, University of Utah, Salt Lake City, Utah, USA; A. Lipton, National Naval Medical Center, Bethesda, Maryland, USA; B. Lothian, Royal University Hospital, Saskatoon, Saskatchewan, Canada; D. Lougheed, Hotel Dieu Hospital, Kingston, Ontario, Canada; B. Lyttle, Children's Hospital of Western Ontario, London, Ontario, Canada; J. Majure, University of Mississippi Medical Center, Jackson, Mississippi, USA; K. Malhotra, Grand River Hospital, Kitchener, Ontario, Canada; J. Marcotte, Hôpital Sainte-Justine, Montreal, Quebec, Canada; T. Martin, Children's Hospital Boston, Boston, Massachusetts, USA; E. Matouk, Montréal Chest Institute, Montreal, Quebec, Canada; M. McCarthy, Providence Medical Center, Spokane, Washington, USA; S. McColley, Ann & Robert H. Lurie Children's Hospital of Chicago, Chicago, Illinois, USA; K. McCoy, Nationwide Children's Hospital, Columbus, Ohio, USA; M. McCubbin, Children's Mercy Hospital, Kansas City, Missouri, USA; K. McDonald, Oregon Health Sciences University, Portland, Oregon, USA; J. McNamara, Children's Hospitals and Clinics of Minnesota, Minneapolis, Minnesota, USA; B. McWilliams, Austin Children's Chest Associates, Austin, Texas, USA; R. Michael, Queen Elizabeth II Health Sciences Centre, Halifax, Nova Scotia, Canada; C. Milla, University of Minnesota, Minneapolis, Minnesota, USA; S. Miller, University of Mississippi Medical Center, Jackson, Mississippi, USA; M. Milot, Centre de Santé et de Services Sociaux de Chicoutimi, Chicoutimi, Quebec, Canada; K. Moffett, West Virginia University, Morgantown, West Virginia, USA; M. Montgomery, Alberta Children's Hospital, Calgary, Alberta, Canada; P. Moore, Vanderbilt University Medical Center, Nashville, Tennessee, USA; A. Moran, University of Minnesota, Minneapolis, Minnesota, USA; W. Morgan, Tucson Cystic Fibrosis Center, Tucson, Arizona, USA; R. Morris, Janeway Children's Health and Rehabilitation Centre, St. John's, Newfoundland and Labrador, Canada; M. Morse, Methodist Children's Hospital, San Antonio, Texas, USA; S. Moskowitz, Children's Hospital & Regional Medical Center, Seattle, Washington, USA; R. Moss, Stanford University Medical Center, Palo Alto, California, USA; P. Murphy, University of Nebraska Medical Center, Omaha, Nebraska, USA; E. Nakielna, St. Paul's Hospital, Vancouver, British Columbia, Canada; S. Nasr, University of Michigan Medical Center, Ann Arbor, Michigan, USA; L. Nassri, Sparks Regional Medical Center, Fort Smith, Arkansas, USA; E. Naureckas, University of Chicago Hospitals, Chicago, Illinois, USA; D. Nielson, University of California at San Francisco, San Francisco, California, USA; M. Noseworthy, Janeway Children's Health and Rehabilitation Centre, St. John's, Newfoundland and Labrador, Canada; B. Noyes, St. Louis University, St. Louis, Missouri, USA; K. Olivier, Wilford Hall United States Air Force (USAF) Medical Center, San Antonio, Texas, USA; E. Olson, University of Florida, Gainesville, Florida, USA; G. Omlor, Akron Children's Hospital, Akron, Ohio, USA; D. Orenstein, Children's Hospital of Pittsburgh, Pittsburgh, Pennsylvania, USA; B. O'Sullivan, University of Massachusetts Memorial Health Care, Worcester, Massachusetts, USA; H.W. Parker, Dartmouth-Hitchcock Medical Center, Lebanon, New Hampshire, USA; M. Passero, Brown University Medical School / Rhode Island Hospital, Providence, Rhode Island, USA; H. Pasterkamp, Children's Hospital of Winnipeg, Winnipeg, Manitoba, Canada; N. Paterson, London Health Sciences Centre, London, Ontario, Canada; L. Pedder, Hamilton Health Sciences Corporation, Hamilton, Ontario, Canada; E. Perkett, Vanderbilt University Medical Center, Nashville, Tennessee, USA; L. Perrault, Centre de Santé et des Services Sociaux de Rouyn-Noranda, Rouyn-Noranda, Quebec, Canada; G. Perry, University of Kansas Medical Center, Kansas City, Kansas, USA; N. Petit, Center Hospitalier Rouyn-Noranda, Rouyn-Noranda, Quebec, Canada; M. Pian, University of California San Diego Children's Hospital, San Diego, California, USA; L. Pineau, Grand River Hospital, Kitchener, Ontario, Canada; A. Platzker, Children's Hospital of Los Angeles, Los Angeles, California, USA; C. Popa, Centre de Santé et des Services Sociaux de Rouyn-Noranda, Rouyn-Noranda, Quebec, Canada; C. Prestidge, Children's Medical Center of Dallas, Dallas, Texas, USA; A. Price, Children's Hospital of Western Ontario, London, Ontario, Canada; D. Quintero, Children's Hospital of Wisconsin, Milwaukee, Wisconsin, USA; L.

Quittell, Columbia University Medical Center, New York, New York, USA; H. Rabin, Foothills Medical Centre, Calgary, Alberta, Canada; P. Radford, Phoenix Children's Hospital, Phoenix, Arizona, USA; F. Ratjen, The Hospital for Sick Children, Toronto, Ontario, Canada; W. Regelmann, University of Minnesota, Minneapolis, Minnesota, USA; C. Ren, University of Rochester Medical Center, Strong Memorial Hospital, Rochester, New York, USA; G. Retsch-Bogart, University of North Carolina at Chapel Hill, Chapel Hill, North Carolina, USA; W. Richards, Memphis Lung Physicians, Southaven, Mississippi, USA; M. Riva, Via-Christi St. Francis, Wichita, Kansas, USA; L. Rivard, Centre Unversitaire de Santé de L'estrie, Sherbrooke, Quebec, Canada; D. Roberts, Providence Medical Center, Anchorage, Alaska, USA; M. Rock, University of Wisconsin Hospital, Madison, Wisconsin, USA; J. Rogers, East Tennessee Children's Hospital, Knoxville, Tennessee, USA; J. Rosen, Albany Medical College, Albany, New York, USA; B. Rosenstein, Johns Hopkins Hospital, Baltimore, Maryland, USA; J. Royall, Children's Hospital of Oklahoma, Oklahoma City, Oklahoma, USA; R. Rubenstein, Children's Hospital of Philadelphia, Philadelphia, Pennsylvania, USA; F. Ruiz, University of Mississippi Medical Center, Jackson, Mississippi, USA; T. Scanlin, Children's Hospital of Philadelphia, Philadelphia, Pennsylvania, USA; M. Schechter, Brown University Medical School / Rhode Island Hospital, Providence, Rhode Island, and Emory University, Atlanta, Georgia, and Wake Forest University Baptist Medical Center, Winston-Salem, North Carolina, USA; H.J. Schmidt, Virginia Commonwealth University, Richmond, Virginia, and National Naval Medical Center, Bethesda, Maryland, USA; P. Schuler, University of Florida, Gainesville, Florida, USA; M. Schwartzman, Joe DiMaggio Children's Hospital, Hollywood, Florida, USA; P. Scott, Georgia Pediatric Pulmonology Associates, PC, Atlanta, Georgia, USA; D. Seilheimer, Baylor College of Medicine, Houston, Texas, USA; G. Shay, Kaiser Permanente Medical Center, Oakland, California, USA; R. Schoumacher, University of Tennessee, Memphis, Tennessee, USA; L. Sicilian, Massachusetts General Hospital, Boston, Massachusetts, USA; R. Simon, University of Michigan Health System, East Lansing, Michigan, USA; P. Smith, Long Island College Hospital, Brooklyn, New York, USA; M. Solomon, The Hospital for Sick Children, Toronto, Ontario, Canada; M. Sontag, University of Colorado Health Sciences Center, Aurora, Colorado, USA; T. Spencer, Children's Hospital of Boston, Boston, Massachusetts, USA; A. Stecenko, Emory University, Atlanta, Georgia, USA; D. Stokes, University of Tennessee, Memphis, Tennessee, USA; B. Sullivan, Marshfield Clinic, Marshfield, Wisconsin, USA; J. Taylor-Cousar, University of New Mexico, Albuquerque, New Mexico, USA; N. Thomas, Pennsylvania State University College of Medicine, Hershey, Pennsylvania, USA; H. Thompson, St. Luke's Cystic Fibrosis Clinic, Boise, Idaho, USA; D. Toder, Children's Hospital of Michigan and Harper University Hospital, Detroit, Michigan, USA; E. Tullis, St. Michael's Hospital, Toronto, Ontario, Canada; N. Turcios, University of Medicine & Dentistry of New Jersey, New Brunswick, New Jersey, USA; R. van Wylick, Hotel Dieu Hospital, Kingston, Ontario, Canada; L. Varlotta, St. Christopher's Hospital for Children, Philadelphia, Pennsylvania, USA; P. Vauthy, Toledo Children's Hospital, Toledo, Ohio, USA; K. Voter, University of Rochester Medical Center, Strong Memorial Hospital, Rochester, New York, USA; J. Voynow, Duke University, Durham, North Carolina, USA; C. Wainwright, Royal Children's Hospital, Melbourne, Victoria, Australia; P. Walker, St. Vincent's Hospital, New York, New York, USA; R. Walker, University of Mississippi Medical Center, Jackson, Mississippi, USA; M. Wall, Oregon Health Sciences University, Portland, Oregon, USA; D. Waltz, Children's Hospital Boston, Boston, Massachusetts, USA; W.S. Warren, Hershey Medical Center, Hershey, Pennsylvania, USA; I. Waters, Royal Jubilee Hospital, Victoria, British Columbia, Canada; P. Wilcox, St. Paul's Hospital, Vancouver, British Columbia, Canada; R. Wilmott, St. Louis University, St. Louis, Missouri, USA; H. Wojtczak, Naval Medical Center, Poway, California, USA; W. Yee, New England Medical Center, Boston, Massachusetts, USA; C. Zacher, St. Alexius Heart & Lung Cystic Fibrosis Clinic, Bismarck, North Dakota, USA; R. Zanni, Monmouth Medical Center, Long Branch, New Jersey, USA; P. Zeitlin, Johns Hopkins Hospital, Baltimore, Maryland, USA;

P. Zuberbuhler, University of Alberta Hospitals, Edmonton, Alberta, Canada; R. Zwerdling, University of Massachusetts Memorial Health Care, Worcester, Massachusetts, USA.
